# Advances in diagnosis of mitochondrial dysfunction and primary mitochondrial disease

**DOI:** 10.1242/dmm.052804

**Published:** 2026-10-01

**Authors:** Melis Kose, Elizabeth McCormick, James T. Peterson, Colleen Muraresku, Angela Viaene, Zarazuela Zolkipli-Cunningham, Marni J. Falk

**Affiliations:** ^1^ Mitochondrial Medicine Frontier Program, Division of Genetic and Genomic Medicine, Department of Pediatrics, Children's Hospital of Philadelphia, Philadelphia, PA 19104, USA; ^2^ Department of Pediatrics, University of Pennsylvania Perelman School of Medicine, Philadelphia, PA 19104, USA; ^3^ Department of Pathology and Laboratory Medicine, Children's Hospital of Philadelphia, Philadelphia, PA 19104, USA; ^4^ Department of Pathology and Laboratory Medicine, University of Pennsylvania Perelman School of Medicine, Philadelphia, PA 19104, USA

**Keywords:** Primary mitochondrial disease, Mitochondrial diagnostics, Mitochondrial disease biomarkers, mtDNA sequencing, Multi-omics

## Abstract

Mitochondria are critical cellular organelles engaged in energy production, diverse intermediary metabolic functions and signaling. Inherited genetic disorders directly affecting mitochondrial structure and/or function, known collectively as primary mitochondrial diseases, are among the most complex and heterogeneous inherited conditions, involving dual-genome origin, multisystem manifestations and widely variable clinical severity. These diseases can follow different inheritance patterns and are caused by pathogenic variants in nearly 400 genes encoded by either the mitochondrial or nuclear genome, which commonly impair proteins or RNAs in distinct molecular pathways, resulting in defective energy production by disrupting oxidative phosphorylation. Mitochondrial diseases are challenging to accurately diagnose because of their extensive clinical variability and common features with other metabolic and neuromuscular disorders, as well as limitations in existing diagnostic tools. However, advances in molecular genetics, imaging and systems biology have transformed the diagnostic landscape, enabling more comprehensive and integrative approaches. This Review provides an overview of mitochondrial physiology and outlines the current state of diagnostic strategies, ranging from conventional biochemical assessments and tissue-based analyses to next-generation genome-wide sequencing and the emerging omics technologies. We discuss how combining classical and modern methods can improve diagnostic accuracy and inform clinical decision making. Additionally, we highlight the need for continued refinement of diagnostic frameworks to better support personalized management and future therapeutic development in mitochondrial medicine.

## Teaser

**Summary:** This Review describes how biomarkers, non-invasive functional testing, tissue-based assays, and genomic and omics approaches are combined to diagnose primary mitochondrial disease in current clinical practice.

## INTRODUCTION

Mitochondria are dynamic organelles for which primary function is the generation of cellular energy in the form of adenosine triphosphate (ATP) via oxidative phosphorylation (OXPHOS; see Glossary) (Falk, 2021). Beyond ATP synthesis, they orchestrate multiple critical cellular processes, including calcium homeostasis, heme and iron–sulfur biosynthesis, apoptosis and the cellular stress response (Rahman and Rahman, 2018). To execute these diverse functions, mitochondria maintain extensive inter-organelle crosstalk, acting as key nodes in intracellular signaling pathways (Russell et al., 2020). According to the MitoCarta 3.0 inventory, the mitochondrial proteome comprises over 1100 proteins under genetic control of both mitochondrial DNA (mtDNA) and nuclear DNA (nDNA) (Rath et al., 2021).

**Table:** 

**Glossary**
**Blue native polyacrylamide gel electrophoresis (BN-PAGE)**
An electrophoretic technique that separates protein complexes isolated from biological membranes, cells or tissues under non-denaturing conditions. This method preserves the native structural integrity of the complexes, allowing for subsequent downstream applications such as in-gel activity assays, electroblotting or immunodetection.
**Cardiopulmonary exercise testing (CPET)**
Integrated whole-body measurement of oxygen uptake, carbon dioxide output and workload during graded exercise. In primary mitochondrial disease (PMD), it can show reduced peak oxygen uptake (peak VO_2_) with an exaggerated cardiovascular response at submaximal workloads, reflecting limited oxidative capacity – a functional finding, not a specific diagnosis.
**Circulating cell-free mitochondrial DNA (ccf-mtDNA)**
Fragments of mitochondrial DNA (mtDNA) released into plasma or cerebrospinal fluid (CSF) from damaged or stressed cells, quantified by droplet digital polymerase chain reaction (PCR) or sequencing.
**Citrate synthase (CS)**
A key nuclear-encoded enzyme that catalyzes the first step of the tricarboxylic acid (TCA) cycle within the mitochondrial matrix. Because its overall activity closely correlates with mitochondrial volume, CS is widely utilized as a robust internal biomarker of cellular mitochondrial mass. Electron transport chain (ETC) enzyme activities are normalized to CS to ensure that any observed diagnostic deficiencies reflect a true defect in specific oxidative phosphorylation (OXPHOS) complexes
**Clark-type oxygen electrode**
A membrane-covered electrochemical sensor that continuously measures dissolved oxygen in a sealed reaction chamber. In respirometry, the decrease in oxygen concentration is used to calculate the oxygen consumption rate of the biological sample.
**Clinical Laboratory Improvement Amendments (CLIA)/College of American Pathologists (CAP) certification**
The US regulatory and accreditation framework – CLIA and CAP certification – under which a laboratory test may be performed and reported for clinical diagnostic use, as distinct from research-use-only assays. Equivalent accreditation frameworks exist in other jurisdictions and are required before a result can guide patient care there.
**Coenzyme Q10 (CoQ10)**
The predominant form of coenzyme Q in humans. It is a lipid-soluble electron carrier within the inner mitochondrial membrane that transfers electrons from complexes I, II and other mitochondrial dehydrogenases to complex III.
**Complexome profiling**
Native gel fractionation coupled to mass spectrometry of sequential slices, giving complex-wide assembly maps.
**Creatine chemical exchange saturation transfer (CrCEST) MRI**
A magnetic resonance imaging (MRI) contrast technique that maps free creatine by selectively saturating its exchangeable protons. Used to track post-exercise creatine recovery kinetics in muscle as a noninvasive surrogate of mitochondrial oxidative capacity. Currently a research tool, not clinically validated.
**Creatine kinase (CK)**
Enzyme that buffers adenosine triphosphate (ATP) by reversible phosphoryl transfer between ATP and creatine in muscle.
**Cytochrome c oxidase (COX)-negative/succinate dehydrogenase (SDH)-positive fibers**
Sequential histochemistry. Cytochrome c oxidase (COX) has three mtDNA-encoded subunits and succinate dehydrogenase (SDH) is entirely nuclear encoded, so loss of COX with retained SDH points to an mtDNA-level defect.
**Digital droplet PCR**
Absolute quantification by droplet partitioning, independent of amplification efficiency. Used where PCR-based next-generation sequencing (NGS) is biased, i.e. deletion heteroplasmy.
**Electron transport chain (ETC)**
Four multi-subunit enzyme complexes (I–IV) embedded in the inner mitochondrial membrane, together with two mobile electron carriers, coenzyme Q and cytochrome c.
**FGF-21 and GDF-15**
Circulating stress-response cytokines induced by mitochondrial dysfunction.
**Heteroplasmy**
The presence of both mutant and wild-type mtDNA molecules within the same cell or tissue, expressed as the percentage of mutant mtDNA. When all mtDNA molecules carry the same sequence, whether wild-type or mutant, this is termed as homoplasmy.
**Lactate**
An end-product of anaerobic glycolysis that serves as a surrogate marker for cytosolic NADH-reductive stress when defective OXPHOS impairs the intracellular NADH:NAD^+^ redox balance. Prolonged tourniquet time, struggling or crying during draw, and delayed sample processing all falsely increase measured lactate and must be excluded before interpreting a result.
**Locus heterogeneity**
A genetic condition in which pathogenic variants in distinct, independent genes result in an identical clinical phenotype.
**Mitochondrial contact site and cristae organizing system (MICOS)**
Inner membrane complex shaping cristae junctions. Loss produces concentric or onion-like cristae on electron microscopy.
**Mitotic segregation**
Random partitioning of mtDNA copies during mitochondrial replication and cell division, combined with selection, causes heteroplasmy levels to drift over time and to diverge between tissues. This is why blood heteroplasmy for m.3243A>G typically declines with age whereas post-mitotic muscle retains or increases mutant load, making blood an unreliable proxy for muscle.
**Mosaicism (fiber level)**
Variable OXPHOS deficiency between adjacent fibers, the histological consequence of the threshold effect. Bulk assays average it out.
**mtDNA copy number**
Number of mtDNA molecules per cell.
**Oxidative phosphorylation (OXPHOS)**
ATP synthesis driven by the proton motive force at complex V. As machinery, it includes complexes I–IV (i.e. ETC) plus ATP synthase.
**Paracrystalline inclusions**
‘Parking-lot’ rectangular crystalline arrays seen on electron microscopy in the mitochondrial intermembrane space between cristae, composed largely of mitochondrial CK. Their presence is suggestive of PMD but not specific to it.
**Pleiotropy**
The genetic phenomenon in which a single gene influences multiple, seemingly phenotypic traits.
**Polarographic assay**
Measures oxygen consumption from the electrical current generated when oxygen is reduced at a polarized electrode. This principle underlies the Clark-type oxygen electrode and Oxygraph-2K respirometry. Requires fresh, intact mitochondria or permeabilized cells – not compatible with frozen tissue.
**Pyruvate dehydrogenase complex deficiency (PDCD)**
Deficiency of the mitochondrial matrix complex that links glycolysis to the TCA cycle, classified as a disorder of substrate utilization/pyruvate metabolism rather than the ETC itself. Frequently grouped with PMD in clinical practice and diagnosed using the same lactate/pyruvate ratio and enzymatic workflows.
**Quadruple immunofluorescence (RC subunit quantification)**
Simultaneous immunofluorescent quantification of complex I and complex IV subunits against markers of mitochondrial mass and cell membrane in single muscle fibers. Provides per-fiber deficiency data more sensitive than standard histochemistry, but is used mainly in specialist research centers rather than routine diagnostics.
**Ragged-red fibers (RRFs)**
Subsarcolemmal accumulation of abnormal mitochondria, red on modified Gomori trichrome.
**Respiratory chain (RC)**
Used in the literature interchangeably with ETC, which includes complexes I–IV.
**Respirometry**
Oxygen consumption as an integrated readout of OXPHOS capacity. A Clark-type oxygen electrode gives a current proportional to dissolved oxygen; state 3 over state 4 defines the respiratory control ratio.
**Seahorse analysis**
Microplate-based measurement of oxygen consumption rate and extracellular acidification rate in intact adherent cells. Sequential injection of mitochondrial inhibitors resolves basal respiration, ATP-linked respiration, proton leak, maximal capacity and spare respiratory capacity. A research assay requiring viable cultured cells, not applicable to frozen tissue.
**Supercomplex**
Higher-order assembly of complexes I, III and IV, resolved as high-molecular-mass bands on BN-PAGE.
**Threshold effect**
A heteroplasmic mtDNA variant causes biochemical or clinical dysfunction only once mutant load exceeds a threshold proportion that is variant- and tissue-specific. This explains why high-energy-demand tissues (brain, muscle, heart) manifest disease first, and why a low heteroplasmy level in blood can coexist with severe disease in muscle.
**Variant of uncertain significance (VUS)**
An American College of Medical Genetics and Genomics (ACMG)/Association for Molecular Pathology (AMP) classification (and the guidelines for classification of genetic sequence variants in clinical testing) applied when the available evidence is either contradictory or insufficient to meet the established criteria for a pathogenic, likely pathogenic, benign, or likely benign designation.

The term ‘mitochondrial disease’ broadly encompasses any genetic and/or acquired conditions that disrupt mitochondrial function and cellular energy homeostasis. Primary mitochondrial diseases (PMDs) are a heterogeneous group of inherited metabolic disorders caused by pathogenic variants in mtDNA or nDNA that directly impair OXPHOS and cellular energy homeostasis (McCormick et al., 2023; Muraresku et al., 2018). Although impaired OXPHOS is the shared hallmark of PMD, the primary genetic defect is not limited to the OXPHOS complexes. PMDs also include disorders affecting pyruvate oxidation, the tricarboxylic acid (TCA) cycle, mtDNA maintenance and expression, cofactor metabolism, protein import, membrane lipid metabolism and mitochondrial dynamics. Currently, pathogenic variants in nearly 400 genes have been established as causative of PMDs, representing one of the primary factors complicating clinical diagnosis (Heath et al., 2025; Messina et al., 2026) (Table S1).

Although PMDs currently represent the most common group of inborn errors of metabolism (IEMs), achieving a definitive clinical diagnosis remains highly complex despite recent technological advancements (Gervasoni et al., 2024). PMDs lack a common clinical presentation; a single gene defect can cause a broad spectrum of phenotypes, while identical clinical phenotypes can arise from variants in many different genes in different mitochondrial pathways. The profound pleiotropy and locus heterogeneity are further complicated by mtDNA heteroplasmy (Fig. 1), which can be explained as the coexistence of mutant and wild-type mtDNA molecules within a single cell or tissue (McCormick et al., 2018, 2023). During cell division, the random partitioning of mtDNA molecules, termed mitotic segregation, can cause heteroplasmy levels to differ among cells and tissues. Biochemical or clinical dysfunction generally occurs only when the mutant load exceeds a variant- and tissue-specific level, known as the threshold effect. Because mutant load can vary significantly across different tissues, establishing clear genotype–phenotype correlations remains a major clinical obstacle, frequently resulting in significant diagnostic delays (Stewart and Chinnery, 2015).

PMD diagnostic approaches have evolved from clinical phenotyping and targeted biochemical testing (Haas et al., 2008) to the use of next-generation sequencing (NGS). NGS-based approaches have markedly improved PMD diagnosis, especially for PMD caused by nDNA variants, and include whole-exome sequencing (WES), whole-genome sequencing (WGS), long-read sequencing methodologies, and transcriptomic and proteomic analyses (Labory et al., 2020). However, although genomic diagnostic testing has increased in recent years, many patients with suspected PMD remain without a definitive molecular diagnosis or are identified with variants of uncertain significance (VUSs). This highlights the need for integrative approaches that combine genomic and multi-omics technologies with assays of mitochondrial function (Falk, 2025; Shen et al., 2020).

In this Review, we describe recent developments in PMD clinical diagnostics and assays that functionally characterize mitochondria. We explore both classical and emerging tools for evaluating mitochondrial dysfunction, ranging from metabolic biomarkers and tissue biopsies to transcriptomic, proteomic and metabolomic profiling, and highlight the translational potential of integrating diagnostic platforms with therapeutic discovery. Our Review provides a comprehensive and clinically relevant framework that reflects the evolving diagnostic landscape of mitochondrial medicine. Accordingly, the Review is organized to reflect the current genomics-first approach to PMD diagnosis, beginning with molecular genetic testing and then addressing biochemical tests, noninvasive functional assessments, tissue-based assays and omics technologies that may be used in parallel or to support the interpretation of molecular findings.

## Molecular diagnosis of PMD

Diagnostic testing for PMD has evolved considerably since mtDNA deletions (Holt et al., 1988) and variants (Wallace et al., 1988a,b) were first reported to be causative of specific PMD phenotypes in 1988, and when the first nDNA gene, *SDHA* (Table S1), was associated with PMD in 1995 (Bourgeron et al., 1995). The discovery of single, PMD-causing genes (DiMauro and Garone, 2010) led to the sequencing of selected genes and of common mtDNA pathogenic variants for clinical purposes. With more recent technological advances in NGS, PMD diagnostic strategies have shifted from a biopsy-first to a genetics-first approach, substantially improving diagnostic yield (McCormick et al., 2013; Wortmann et al., 2015). Nevertheless, targeted NGS diagnostic strategies have limited success in diagnosing patients with suspected PMD caused by as-yet uncharacterized genes or variants with tissue-specific expression patterns (Ng et al., 2025; Stenton and Prokisch, 2020). As a result, molecular diagnostics increasingly incorporate comprehensive genomic and functional analyses, including mtDNA copy number assessment, heteroplasmy quantification, transcriptomics and, more recently, proteomics to support variant classification (Hock et al., 2025; Kremer et al., 2017). In this section, we review the evolving role of molecular diagnostic strategies in the evaluation of suspected PMD (Fig. 2).

**Fig. 1.**
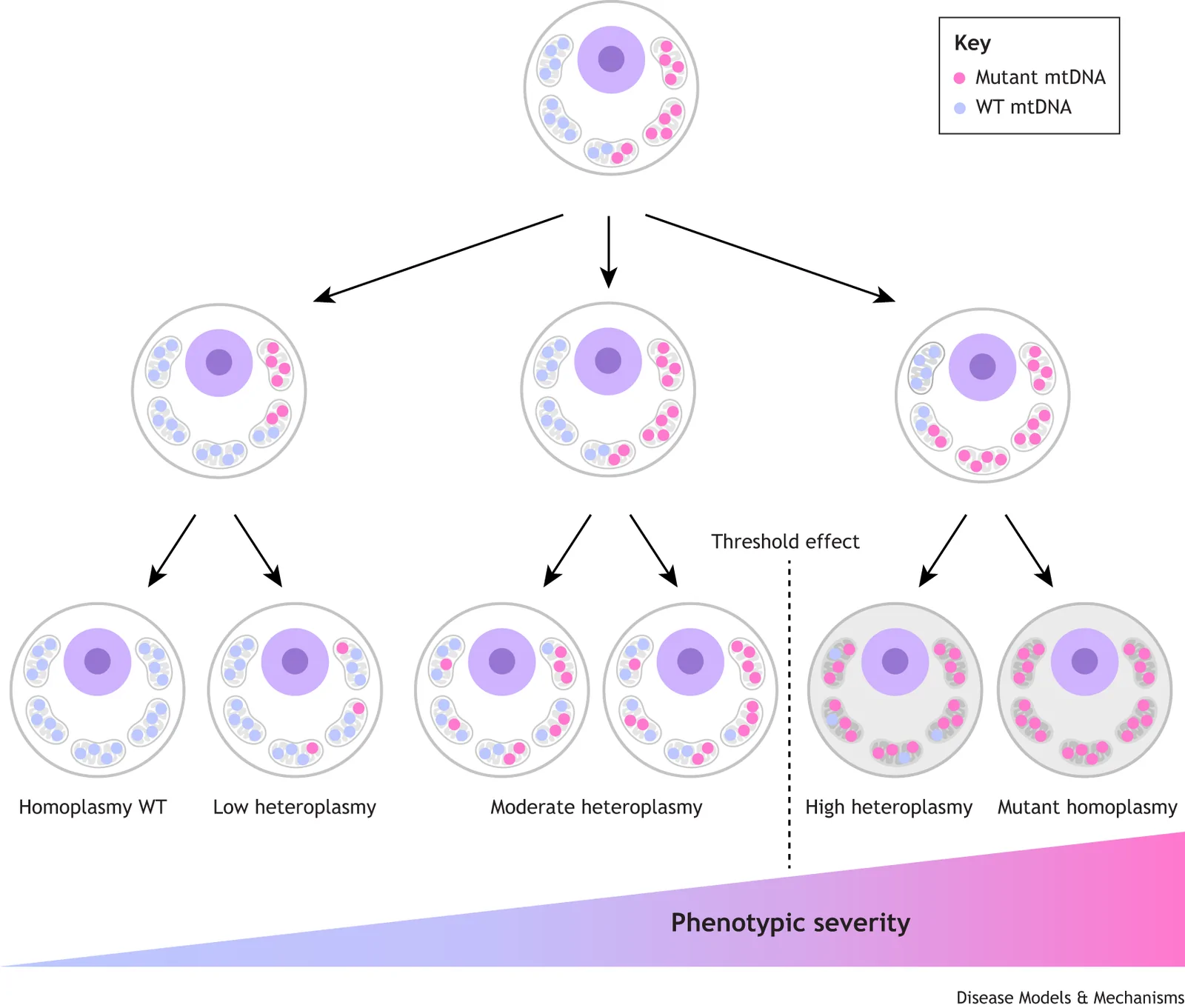
**Mitotic segregation and the threshold effect of mtDNA heteroplasmy.** Heteroplasmy is defined as the coexistence of both mutant (pink) and WT (blue) mtDNA molecules within a single cell or tissue. During cell division, the random partitioning of mtDNA copies, termed mitotic segregation, causes heteroplasmy levels to drift, resulting in daughter cells with highly variable proportions of mutant mtDNA. This distribution ranges from pure WT homoplasmy to low, moderate and high heteroplasmy, ultimately reaching mutant homoplasmy when all mtDNA molecules carry the specific variant sequence. A pathogenic heteroplasmic mtDNA variant typically causes clinical or biochemical dysfunction only once the mutant load exceeds a critical proportion, a phenomenon known as the threshold effect (represented in the figure by the dashed line). As the proportion of mutant mtDNA surpasses this tissue- and variant-specific threshold, phenotypic severity progressively increases. mtDNA, mitochondrial DNA; WT, wild type.

**Fig. 2.**
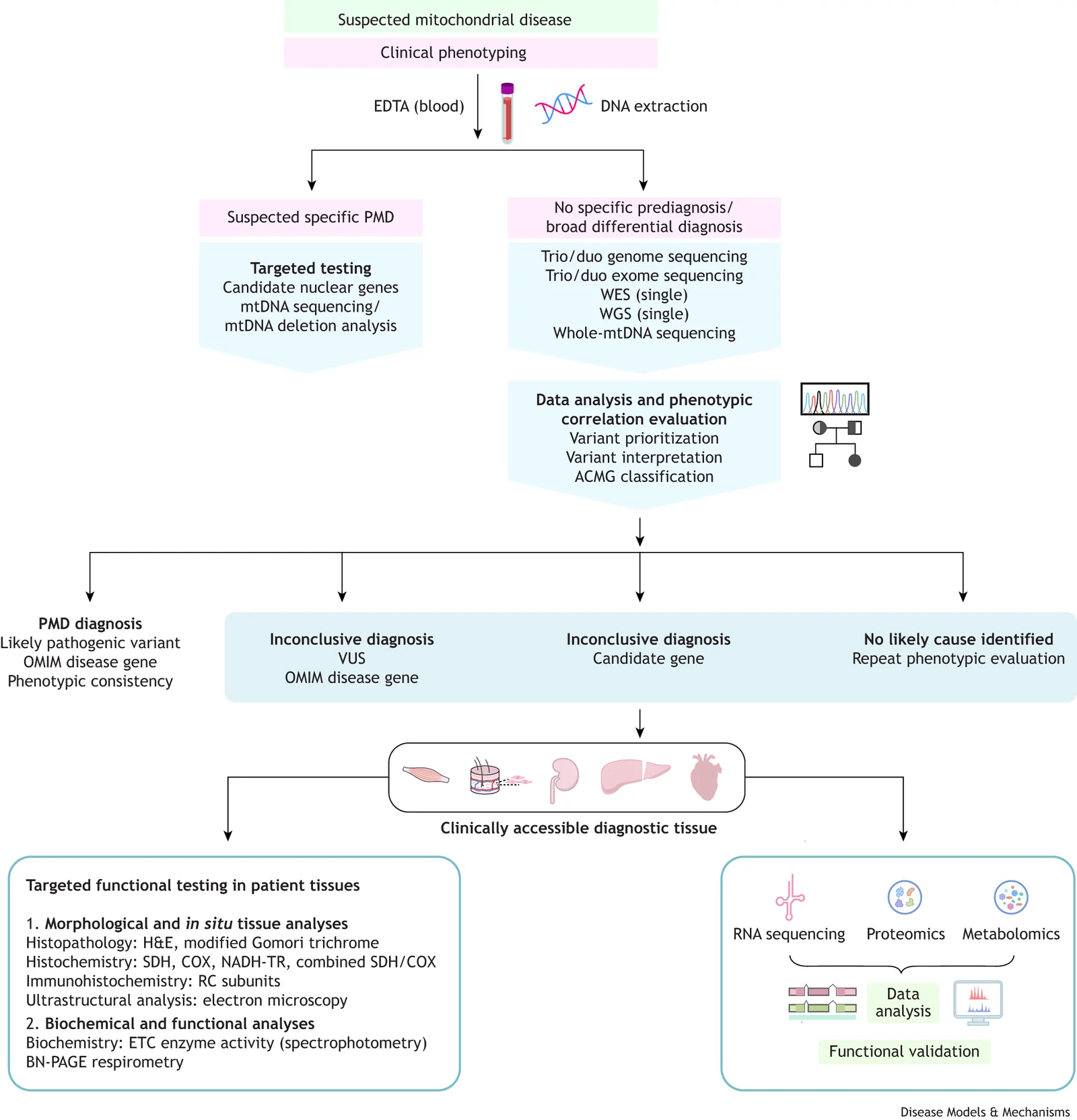
**Genomics-first diagnostic approach for suspected primary mitochondrial disease.** Clinical phenotyping guides the selection and interpretation of molecular testing. When a specific PMD is suspected, testing may be directed toward candidate nuclear genes or include mtDNA sequencing and deletion analysis. When there is no specific diagnostic hypothesis, broader testing may include exome or genome sequencing, preferably with parental samples when available (trio) or with affected sibling (duo), together with complete mtDNA sequencing. Identified variants are prioritized, classified according to ACMG criteria, and evaluated in relation to the phenotype and expected inheritance pattern. A molecular diagnosis may be established when pathogenic or likely pathogenic variant(s) in an established disease gene are consistent with these findings. A VUS in an established disease gene or a potentially relevant variant in a candidate gene requires further evaluation. In such cases, a clinically relevant and accessible tissue may be selected for morphological, histochemical, biochemical and functional analyses. RNA sequencing, proteomics and metabolomics may be performed in parallel or considered when targeted studies remain inconclusive. If no likely genetic cause is identified, phenotypic and genomic findings may be reassessed and additional tissue-based or omics analyses considered. These investigations provide functional evidence for evaluating the relationship between the identified variant and mitochondrial dysfunction. ACMG, American College of Medical Genetics and Genomics; BN-PAGE, blue native polyacrylamide gel electrophoresis; COX, cytochrome c oxidase; EDTA, ethylenediaminetetraacetic acid; ETC, electron transport chain; H&E, Hematoxylin and Eosin; mtDNA, mitochondrial DNA; NADH-TR, nicotinamide adenine dinucleotide–tetrazolium reductase; OMIM, Online Mendelian Inheritance in Man; PMD, primary mitochondrial disease; RC, respiratory chain; SDH, succinate dehydrogenase; VUS, variant of uncertain significance; WES, whole-exome sequencing; WGS, whole-genome sequencing.

### mtDNA genome sequencing, deletion and copy number analysis

mtDNA mutation testing has evolved significantly over the past two decades. In the mid-2000s, sequencing the full mtDNA genome via the polymerase chain reaction (PCR)-based Sanger sequencing method became clinically available and was widely adopted as the standard of care (McCormick et al., 2013; Taylor and Turnbull, 2005). However, this approach had limitations, particularly concerning the reliable detection of mutations at heteroplasmy levels below 30–50% (McCormick et al., 2013). Prior to the use of sequencing, traditional methods, such as PCR-restriction fragment length polymorphism, long-range PCR and Southern blotting were used to screen for a panel of ∼10–12 mtDNA common variants and deletions. However, these techniques also lacked sensitivity when assaying low-level heteroplasmic variants (McCormick et al., 2013). New techniques address these limitations by making use of long-range PCR enrichment in combination with NGS, enabling both qualitative and quantitative evaluation of the entire mtDNA genome. Importantly, NGS-based platforms enable the concurrent identification of point mutations, large-scale deletions and other structural variants, such as duplications, in a single assay. These platforms can reliably detect heteroplasmy levels as low as 1–10%, significantly improving diagnostic resolution compared to these earlier techniques (Stenton and Prokisch, 2020; Wang et al., 2022). The selection of tissue for mtDNA sequencing and deletion analysis should be guided by the patient's age, phenotype and suspected type of mtDNA abnormality. Blood is generally informative in children, although variants restricted to skeletal muscle may be missed. In adults, urine sediment or skeletal muscle can provide greater diagnostic sensitivity, particularly for mtDNA variants or rearrangements that are present at low or undetectable levels in blood. Urine sediment contains renal epithelial cells with a high mitochondrial content and, therefore, provides a noninvasive and informative alternative to muscle for mtDNA analysis (Wen et al., 2025).

Both single large-scale mtDNA deletions (SLSMDs) and multiple large-scale mtDNA deletions (MLSMDs) can be detected during the evaluation of PMD. A single large-scale mtDNA deletion can establish a molecular diagnosis of an SLSMD, whereas MLSMDs may occur as a consequence of pathogenic variants in over 20 nuclear genes that are responsible for mtDNA maintenance, commonly including *POLG* or *RRM2B* (Table 1), or that occur due to aging or environmental factors (Alston et al., 2021). Differentiating between SLSMDs and MLSMDs is essential for accurate molecular diagnosis and downstream clinical management. In practice, many diagnostic laboratories use a total deletion heteroplasmy threshold of >10% when reporting MLSMDs (Wang et al., 2022, 2025).

**Table 1.** Classification and key clinical features of disorders discussed in this Review

**Disorder**	**Causative gene(s)**	**Classification regarding PMD**	**Key clinical features**	**Section in the Review**
Alpers syndrome (Alpers–Huttenlocher syndrome)	*POLG*	PMD	Intractable epilepsy, developmental regression and hepatopathy in infancy or early childhood; valproate is contraindicated because of hepatotoxicity.	‘Genotype–phenotype correlations: predicting disease progression and outcomes’ – cited as an example of one *POLG* variant producing different phenotypes
Barth syndrome	*TAZ*	PMD	Cardiomyopathy, skeletal myopathy, cyclic neutropenia and growth delay in males; 3-methylglutaconic aciduria on urine organic acid analysis.	‘Urine organic acid quantitative analysis’ –cited as a cause of 3-methylglutaconic aciduria
CoQ10 deficiency, primary	*COQ2*, *COQ4*, *COQ5*, *COQ6*, *COQ7*, *COQ8A* (*ADCK3*), *COQ8B*, *COQ9*, *PDSS1*, *PDSS2*	PMD	Highly variable: steroid-resistant nephrotic syndrome, cerebellar ataxia, encephalomyopathy or isolated myopathy.	‘Coenzyme Q10 determination’ – muscle CoQ10 quantification by HPLC/LC-MS/MS
CPEO	SLSMD spectrum; also *POLG* and other nuclear mtDNA-maintenance genes	PMD	Ptosis and progressive limitation of eye movements, often with proximal myopathy and exercise intolerance; typically, the latest and mildest stage of the SLSMD spectrum.	‘Genotype–phenotype correlations: predicting disease progression and outcomes’ – the SLSMD phenotypic continuum
Ethylmalonic encephalopathy	*ETHE1*	PMD	Early-onset encephalopathy, petechiae, chronic diarrhea, acrocyanosis and ethylmalonic aciduria.	‘Urine organic acid quantitative analysis’ – ethylmalonic acid listed among non-specific elevations
Fumarase deficiency	*FH*	PMD	Neonatal encephalopathy, severe developmental delay, brain malformations and fumaric aciduria.	‘Urine organic acid quantitative analysis’ – cited among disorders of TCA flux that mimic PMD
KSS	SLSMD	PMD	Onset before age 20 with CPEO, pigmentary retinopathy and at least one of cardiac conduction block, cerebellar ataxia or raised CSF protein; short stature and endocrinopathy are common. Choroid plexus involvement lowers CSF 5-methyltetrahydrofolate.	‘Cerebrospinal fluid biomarker biochemistry’, ‘5-methyltetrahydrofolate’; ‘Genotype–phenotype correlations: predicting disease progression and outcomes’
Leigh syndrome spectrum	114 curated gene–disease associations (nuclear and mtDNA), e.g. *SURF1*, *NDUFS4*, *MT-ATP6*	PMD	Subacute developmental regression, hypotonia, brainstem and basal ganglia T2 hyperintensities on MRI, with lactate elevation in blood and/or CSF. The most common pediatric PMD presentation.	‘Cerebrospinal fluid biomarker biochemistry’, ‘Lactate’; acyl-carnitine profiles; ClinGen gene–disease curation
Lipoate synthesis defect (variant nonketotic hyperglycinemia)	LIAS; related: *BOLA3*, *GLRX5*, *IBA57*, *LIPT1*, *LIPT2*, *NFU1*	PMD	Neonatal-onset encephalopathy, seizures and lactic acidosis with raised glycine (hence ‘variant nonketotic hyperglycinemia’), distinguishing it from classical NKH by the accompanying lactic acidosis and multiple enzyme deficiencies.	‘Urine organic acid quantitative analysis’ – cited with impaired glycine cleavage
Maple syrup urine disease and branched-chain aminoacidurias	*BCKDHA*, *BCKDHB*, *DBT*	Organic aciduria/aminoacidopathy	Neonatal encephalopathy with maple-syrup odor and raised branched-chain amino acids; secondary lactate elevation can mimic PMD.	‘Urine organic acid quantitative analysis’ – cited as an IEM that mimics PMD
MELAS	*MT-TL1*	PMD	Stroke-like episodes not conforming to vascular territories, encephalopathy, seizures, lactic acidosis, sensorineural hearing loss and short stature; low plasma citrulline and arginine. Low heteroplasmy of the same variant more often presents as MIDD.	‘Urine organic acid quantitative analysis’; circulating cell-free mtDNA rises further during stroke-like episodes, changing heteroplasmy levels during aging
MERRF	*MT-TK* (*m.8344A>G*)	PMD	Myoclonus, generalized epilepsy, cerebellar ataxia and myopathy with ragged-red fibers on muscle histology; markedly elevated GDF-15.	‘GDF-15’ – second-highest levels after SLSMD disorders
Mitochondrial DNA depletion syndromes	*POLG*, *TWNK*, *TK2*, *DGUOK*, *MPV17*, *RRM2B*, *SUCLA2*, *SUCLG1*, *TFAM*, *SLC25A4* and others	PMD	Tissue-specific presentations: hepatocerebral (DGUOK, MPV17), myopathic (TK2), encephalomyopathic with methylmalonic aciduria (SUCLA2, SUCLG1) and neurogastrointestinal (TYMP).	N/A
MRPS34-related combined OXPHOS deficiency	*MRPS34*	PMD	Leigh syndrome phenotype with developmental delay, hypotonia, lactic acidosis and combined respiratory chain deficiency.	‘Omics technologies in diagnosis of PMD’ – cited as a gene for which variant pathogenicity was validated by proteomics
MT-ATP6-related mitochondrial disease	*MT-ATP6*	PMD	Phenotype depends on heteroplasmy level and ranges from Leigh syndrome at high levels to NARP at lower levels; low citrulline may be detected on newborn screening.	‘Urine organic acid quantitative analysis’ – low citrulline on newborn screening; acyl-carnitine profiles
NDUFAF8-related complex I deficiency	*NDUFAF8*	PMD	Leigh syndrome phenotype with early-onset encephalopathy and isolated complex I deficiency in muscle and fibroblasts.	‘Omics technologies in diagnosis of PMD’ – variant pathogenicity validated by proteomics
Pearson syndrome	SLSMD	PMD	Infantile sideroblastic anemia with vacuolated marrow precursors and exocrine pancreatic insufficiency; survivors may evolve to KSS and then CPEO.	‘GDF-15’; ‘Genotype–phenotype correlations: predicting disease progression and outcomes’ – the SLSMD continuum
PDCD	*PDHA1* (X-linked), *PDHB*, *DLAT*, *PDHX*, *PDP1*, *DLD*	PMD	Neonatal or infantile lactic acidosis, hypotonia, developmental delay and structural brain malformations; a normal lactate-to-pyruvate ratio despite raised lactate distinguishes it from respiratory chain disease.	‘Plasma lactate and pyruvate’ – the lactate-to-pyruvate ratio distinguishes PDCD from OXPHOS disorders (blood and CSF)
POLG-related disorders	*POLG*	PMD	A continuum by age of onset: Alpers syndrome in early childhood (worst prognosis), ataxia–neuropathy and epilepsy in juvenile/adult onset, and ptosis with PEO in late onset. The most prevalent childhood mtDNA maintenance defect.	‘Genotype–phenotype correlations: predicting disease progression and outcomes’ – identical variants producing Alpers or PEO
Secondary cerebral folate deficiency	Not monogenic in this context – secondary to PMD	A secondary consequence of PMD	Low CSF 5-methyltetrahydrofolate with otherwise unexplained neurological deterioration.	‘Cerebrospinal fluid biomarker biochemistry’, ‘5-methyltetrahydrofolate’
SLSMD spectrum	SLSMD	PMD	Progressive multi-system involvement over the lifespan, from infantile bone marrow failure and pancreatic insufficiency through the KSS triad to adult CPEO.	‘GDF-15’; ‘Genotype–phenotype correlations: predicting disease progression and outcomes’
Succinate-coenzyme A ligase deficiency	*SUCLA2*, *SUCLG1*	PMD	Encephalomyopathy with dystonia, sensorineural hearing loss and mild methylmalonic aciduria; mtDNA depletion in muscle.	‘Urine organic acid quantitative analysis’ – currently cited as a TCA-flux mimic
TIMMDC1-related complex I deficiency	*TIMMDC1*	PMD	Infantile-onset encephalomyopathy with isolated complex I deficiency, muscular hypotonia and developmental delay.	‘Omics technologies in diagnosis of PMD’ – variant pathogenicity validated by multi-omics

CoQ10, coenzyme Q10; CPEO, chronic progressive external ophthalmoplegia; CSF, cerebrospinal fluid; HPLC, high-performance liquid chromatography; IEM, inborn errors of metabolism; KSS, Kearns–Sayre syndrome; LC-MS/MS, liquid chromatography–tandem mass spectrometry; LIAS, lipoic acid synthetase; MELAS, mitochondrial encephalomyopathy, lactic acidosis and stroke-like episodes; MERRF, myoclonic epilepsy with ragged-red fibers; MIDD, maternally inherited diabetes and deafness; MRI, magnetic resonance imaging; mtDNA, mitochondrial DNA; N/A, not applicable; NARP, neuropathy, ataxia, retinitis pigmentosa; NKH, non-ketotic hyperglycinemia; OXPHOS, oxidative phosphorylation; PDCD, pyruvate dehydrogenase complex deficiency; PEO, progressive external ophthalmoplegia; PMD, primary mitochondrial disease; SLSMD, single large-scale mtDNA deletion; TCA, tricarboxylic acid.

When interpreting molecular tests for mtDNA-related PMD, it is important to recognize that heteroplasmy levels can change with age and may follow different patterns across tissues (Grady et al., 2018). The exact mechanisms are not fully understood and may differ according to the type and pathogenicity of the mtDNA variant and whether the tissue is mitotic or postmitotic. A well-known example of pathogenic point mutations is the m.3243A>G mutation in the *MT-TL1* (Table 1) gene, which generally decreases in blood over a lifetime (Pickett et al., 2018). This decline is thought to reflect negative selection against blood-cell precursors with high variant levels, as their impaired function or reduced proliferative capacity limits their contribution to the circulating blood-cell population over time (Filograna et al., 2021). mtDNA deletions can follow a different pattern. Deleted mtDNA molecules are smaller than wild-type mtDNA molecules and may replicate more rapidly, allowing them to accumulate in postmitotic tissues such as skeletal muscle. Blood heteroplasmy for m.3243A>G generally decreases with age; deletion heteroplasmy may increase in postmitotic tissues. The heteroplasmy level measured in a clinical sample therefore depends on the mtDNA variant, the tissue tested and the patient's age, and the sample should be selected accordingly (Stewart and Chinnery, 2015). Although mtDNA sequencing of blood is generally sensitive in pediatric patients, it yields low diagnostic sensitivity for detecting mtDNA rearrangements and copy number defects in adults. A normal blood result in an adult does not rule out PMD, especially for variants restricted to muscle. Diagnostic sensitivity in adults is much greater when utilizing urine sediment or skeletal muscle. Additionally, when downstream functional or molecular analyses require cell culture, pathogenic variants can be lost while passaging primary fibroblasts (Filograna et al., 2021).

Another critical parameter in the diagnosis of PMD is the measurement of mtDNA content. mtDNA content quantification is a common assessment to identify mtDNA depletion. Causes of primary mtDNA depletion must be confirmed through nuclear genome diagnostic testing of several dozen different genes, which commonly include mtDNA maintenance disorders such as those caused by pathogenic variants in *POLG*, *RRM2B*, *DGUOK* and *TWNK*, among others (El-Hattab et al., 2017). Similar to mitochondrial RC enzymatic activity analysis, nutritional status and current medications should be taken into consideration when evaluating the etiology of reduced mtDNA content (Mohamed Yusoff et al., 2025). Conversely, proliferation of mtDNA content can also be seen as an adaptive change in PMD (Torres-Odio et al., 2021), including commonly in Leber's hereditary optic neuropathy and mitochondrial transfer RNA (MT-tRNA) disorders. Recent work also reveals that mtDNA copy number alterations are associated with altered cellular bioenergetics and metabolic flexibility in contexts of primary genetic defects and secondary mitochondrial stress (Mohamed Yusoff et al., 2025). These findings underscore the necessity for genomic diagnostics to not only identify primary causative variants but also distinguish features of secondary mitochondrial dysfunction, where mtDNA proliferation may modulate disease phenotypes (Filograna et al., 2021; Torres-Odio et al., 2021). mtDNA content analysis in muscle or liver testing is available at several clinical diagnostic laboratories in the USA at the present time (Table 2).

**Table 2.** CLIA-certified and CAP-accredited US laboratories with mitochondrial biomarker/functional assessment assays

Test		Performing Lab	Tissue	
BN-PAGE of OXPHOS complexes		Colorado Children's Hospital	Skin fibroblasts	https://childrenscolorado.testcatalog.org/show/LAB7078
			Skeletal muscle	https://childrenscolorado.testcatalog.org/show/LAB7087
			Heart	https://childrenscolorado.testcatalog.org/show/LAB7087
			Liver	https://childrenscolorado.testcatalog.org/show/LAB7087
Coenzyme Q10 determination		Children's Hospital of Philadelphia	Leukocytes	https://www.testmenu.com/chop/Tests/1107388
		MNG (Labcorp)	Leukocytes	https://mnglabs.labcorp.com/tests/620043/coenzyme-q10-leukocytes
			Skeletal muscle	https://mnglabs.labcorp.com/tests/MET05/coenzyme-q10-muscle
Complex I western blot analysis		Colorado Children's Hospital	Skin fibroblasts	https://childrenscolorado.testcatalog.org/show/LAB9301
Mitochondrial respiratory chain/electron transport chain analysis		Baylor Genetics	Skin fibroblasts	https://catalog.baylorgenetics.com/details/3210
			Skeletal muscle	https://catalog.baylorgenetics.com/details/3200
		Colorado Children's Hospital	Skin fibroblasts	https://childrenscolorado.testcatalog.org/show/LAB7087
			Skeletal muscle	https://childrenscolorado.testcatalog.org/show/LAB7086
			Heart	https://childrenscolorado.testcatalog.org/show/LAB7086
			Liver	https://childrenscolorado.testcatalog.org/show/LAB7086
GDF-15		Mayo Clinic	Plasma	https://www.mayocliniclabs.com/test-catalog/overview/64637
		Colorado Children's Hospital	Serum/plasma	https://childrenscolorado.testcatalog.org/show/LAB10330
FGF-21		Colorado Children's Hospital	Serum/plasma	https://childrenscolorado.testcatalog.org/show/LAB10330
Glutathione	Total	Labcorp	Whole blood	https://www.testmenu.com/chop/Tests/1221887
	Total Reduced Oxidized	Children's Hospital of Philadelphia	Whole blood	https://www.labcorp.com/tests/007700/glutathione-total#test-specimen
		Stanford Medicine	Whole blood	https://stanfordlab.com/content/stanfordlab/en/test-details/l/LAB274.html
Ketone body panel (beta-hydroxybutyrate to acetoacetate)		Children's Hospital of Philadelphia	Serum	Children's Hospital of Philadelphia Test Directory | Tests
Plasma amino acid analysis		Children's Hospital of Philadelphia	Plasma	Metabolic and Advanced Diagnostics | Children's Hospital of Philadelphia
		Mayo Clinic	Plasma	Amino Acids, Quantitative, Plasma – Mayo Clinic Laboratories | Neurology Catalog
		Labcorp	Plasma	700068: Amino Acid Profile, Quantitative, Plasma | Labcorp
Mitochondrial DNA content		Baylor Genetics	Skeletal muscle	https://catalog.baylorgenetics.com/details/3700
			Liver	https://catalog.baylorgenetics.com/details/3720
		Children's Hospital of Philadelphia	Skeletal muscle	https://www.testmenu.com/chop/Tests/1142797
Respirometry (OXPHOS polarographic analysis)		Children's Hospital of Philadelphia	Skin fibroblasts	https://www.testmenu.com/chop/Tests/1009395

BN-PAGE, blue native polyacrylamide gel electrophoresis; CAP, College of American Pathologists; CLIA, Clinical Laboratory Improvement Amendments; OXPHOS, oxidative phosphorylation.

### NGS and associated technologies

NGS technologies have enabled multiple candidate genetic etiologies to be simultaneously evaluated in PMD. This advance has been especially important for those with suspected PMD, given the nonspecific, multi-system nature of PMDs’ many clinical features and their overlap with other genetic disorders (McCormick et al., 2013; Wortmann et al., 2015) (Fig. 2). NGS can be performed independently or with targeted capture together with WES, and can reliably provide information on mtDNA variant heteroplasmy levels (Falk et al., 2012; Stenton and Prokisch, 2020). NGS can also detect large-scale mtDNA deletions, although, as discussed above, SLSMD or MLSMD heteroplasmy levels cannot be accurately quantified by NGS because the PCR amplification step selectively over-represents the smaller mtDNA genome molecules that harbor deletions. For this reason, additional non-PCR-based analytic technologies such as digital droplet PCR are needed to accurately quantify levels of deletion heteroplasmy (Wang et al., 2022; Ganetzky et al., 2025).

WES, involving library-based capture and sequencing of exons from more than 20,000 nuclear genes, became clinically available in the early 2010s and was broadly considered the first-tier clinical diagnostic test for PMD by the mid-2010s (Muraresku et al., 2018). WES made it possible to simultaneously sequence parental and/or other family members' samples and to use filtering algorithms to evaluate variant segregation, thereby improving diagnostic efficiency and reducing the VUS candidate number. However, WES is limited in that it enables sequencing of only 2% of the genome – the ‘exome’ coding regions. In addition, WES achieves comprehensive sequence coverage for only ∼98% of the captured exome. These limitations are reflected in studies that report WES diagnostic rates ranging from 35% to 70%, depending on the specific PMD phenotype (Stenton and Prokisch, 2020).

WGS is increasingly available in clinical diagnostic laboratories and may include analysis of both the nuclear and mitochondrial genomes. WES and WGS have both historically been performed by short-read sequencing (which fragments DNA into 100–300-bp segments that are then computationally assembled, annotated and analyzed). By contrast, WGS provides comprehensive sequence coverage of the ‘exome’ without requiring a library capture step. Although short-read WGS can detect sequence variants in intronic regions and triplet repeat expansions, which WES cannot, studies have shown that the clinical diagnostic rates achieved with WGS are not dramatically increased compared to those obtained with WES (Ng et al., 2025; Stranneheim et al., 2021). Long-read WGS (LR-WGS; which sequences genomic fragments of 1 kb to >2 Mb) is emerging as a promising technology available on a research basis that is just beginning to come online in clinical diagnostic laboratories. LR-WGS offers several technological benefits, including (1) the ability to phase variants in a proband's genome without requiring parental samples (although parental samples are still required to determine whether a variant is *de novo* or inherited); (2) increased sensitivity in detecting structural rearrangements; (3) improved ability to quantify triplet repeat expansions throughout the genome; (4) the ability to detect methylation; and (5) excellent coverage of large fragments or of the entire mtDNA genome without requiring additional capture or amplification technology (Eisfeldt et al., 2025). Given these comprehensive improvements and rapidly falling cost, the genomics community is considering the use of LR-WGS as a first-tier diagnostic test for complex disorders, including PMD. However, challenges exist, most notably regarding the lack of widespread clinical test availability, cost and insurance coverage, and the ability to interpret the significance of reported variants in non-genic regions (Macken et al., 2023; Slapnik et al., 2024). Especially for the interpretation challenges, mtDNA variants present an additional difficulty that requires more attention. Specifically, the high sensitivity of NGS platforms introduces clinical nuances regarding mtDNA heteroplasmy (Schon et al., 2021). It is essential to correlate any identified pathogenic variant and its heteroplasmy level with both the specific tissue tested and the patient's clinical presentation (McCormick et al., 2020). Emerging evidence suggests that the broad application of NGS is increasingly picking up low-level variants that may represent incidental findings or asymptomatic carrier status rather than the primary disease etiology (Stewart and Chinnery, 2015). Because heteroplasmy varies significantly across tissues, a variant detected at low levels in blood may lack clinical relevance if it does not align with the patient's phenotype or the mutant load in clinically affected tissues (Craven et al., 2017). Consequently, multidisciplinary evaluation is required to appropriately manage incidental findings and ensure that the detected genomic variants are truly causative of the phenotype.

Molecular genetic testing is now the first-line approach for diagnosing suspected PMD and includes analysis of both the mitochondrial and nuclear genomes. mtDNA sequencing, deletion and copy-number analysis require careful selection of the tested tissue because heteroplasmy levels and the detection of mtDNA abnormalities can vary with age and among tissues. Nuclear gene panels, WES, WGS and emerging long-read sequencing methods enable broader detection of PMD-causing variants, although limitations in sequence coverage and variant interpretation remain. Available databases and community resources play a crucial role in advancing PMD diagnostics (Box 1). Identified variants must be evaluated in relation to the patient's phenotype, inheritance pattern, tissue distribution, and available population and disease-specific data, and many patients still receive an inconclusive result or a VUS. In these cases, biochemical testing can provide complementary evidence of mitochondrial dysfunction and support the interpretation of molecular findings.

Box 1. Resources for variant interrogation Databases and community resources facilitate diagnostic discovery and our understanding of rare diseases, including primary mitochondrial disease (PMD). Our knowledge of variant allele frequency in presumably healthy population-scale databases has grown over time (Chen et al., 2024), along with coordinated efforts to systematically review gene–disease associations (Strande et al., 2017) and variant pathogenicity (McCormick et al., 2020; Richards et al., 2015). Two Curation Expert panels have been created specifically for PMD, ClinGen-approved Mitochondrial Diseases Gene and Nuclear and Mitochondrial Variant. These panels involve global collaborations among mitochondrial disease clinicians, diagnostic laboratories and researchers to undertake rigorous protocols to establish gene–disease and variant–disease associations for many forms of PMD, as well as actionable guidelines for gene and variant curation. This work has included the expert curation of 114 gene–disease associations for Leigh syndrome spectrum (McCormick et al., 2023), focusing on published genes and variants listed in the public ClinVar database. The American College of Medical Genetics (ACMG) and Association of Molecular Pathology (AMP) guidelines on variant interpretation have also been reviewed and specified for mitochondrial DNA (mtDNA) variant interpretation (McCormick et al., 2020; Richards et al., 2015). Additional general genomics community informatics resources include Gene Matcher (Sobreira et al., 2015) and Variant matcher (Wohler et al., 2021). The Mitochondrial Disease Sequence Data Resource (MSeqDR) is another important PMD community resource. This is a centralized, publicly accessible Web resource that hosts both nuclear DNA (nDNA) and mtDNA genomic and phenotypic information sources and analytic tools (Falk et al., 2015). MSeqDR was established in 2012 by a grass-roots consortium of mitochondrial disease clinicians, researchers, information, and laboratory experts with United Mitochondrial Disease Foundation (UMDF) partnership and support. It includes links to the MITOMAP curated database of mtDNA variants in disease and populations (Lott et al., 2013) and provides compiled gene and sequence information from population databases, *in silico* prediction tools, *in silico* raw exome or genome data analytic tools, PMD phenotype ontology trees and descriptions, as well as manual literature reviews for mtDNA variants, and links to mtDNA and nDNA gene variants in all known and candidate mitochondrial disease genes (Falk, 2020). MSeqDR also hosts the Quick-Mitome analytic tool to enable user-based, phenotype-guided raw sequence data analysis of proband and kindred exomes and genomes to aid PMD diagnostic discovery across every potential inheritance pattern, as well as a searchable PMD virtual registry of thousands of PMD cases curated from the published literature (Shen et al., 2024). A host of other tools exist to aid this effort, including the mitoPhen database of presumed mitochondrial disease-associated mtDNA variants, ontology terms and publications (Ratnaike et al., 2021), and a mtDNA variants visualization resource mitoVisualize (Lake et al., 2022). Efforts also continue to improve mtDNA *in silico* variant predictor tools, such as APOGEE (Bianco et al., 2023). Several resources also exist for nDNA disorders, most notably for *POLG*-related PMD, including the *POLG* database and *POLG* server (Nurminen et al., 2017) for POLG-related disorders.

## Biochemical evaluation of mitochondrial function

This section highlights biochemical testing of blood, urine and cerebrospinal fluid (CSF) in the diagnosis of PMD. These diagnostic tools complement other approaches and genomic testing, particularly when used in parallel to support, refine and strengthen the interpretation of genetic test results in patients with suspected PMD (Fig. 3). We focus here on diagnostic tests that are currently clinically available in the USA, together with emerging analytes that remain under investigation. Selected clinically available assays for evaluating mitochondrial function, which are performed in laboratories with Clinical Laboratory Improvement Amendments (CLIA)/College of American Pathologists (CAP) certification, are summarized in Table 2 (the availability of equivalent diagnostic tests and specialized centers across Europe can be found on the website of European Reference Network for Mitochondrial Disorders). Although the diagnostic performance of these tests has been evaluated to varying degrees, evidence supporting their use to quantify disease severity, monitor progression or assess response to therapeutic interventions in PMD remains limited and test specific.

**Fig. 3.**
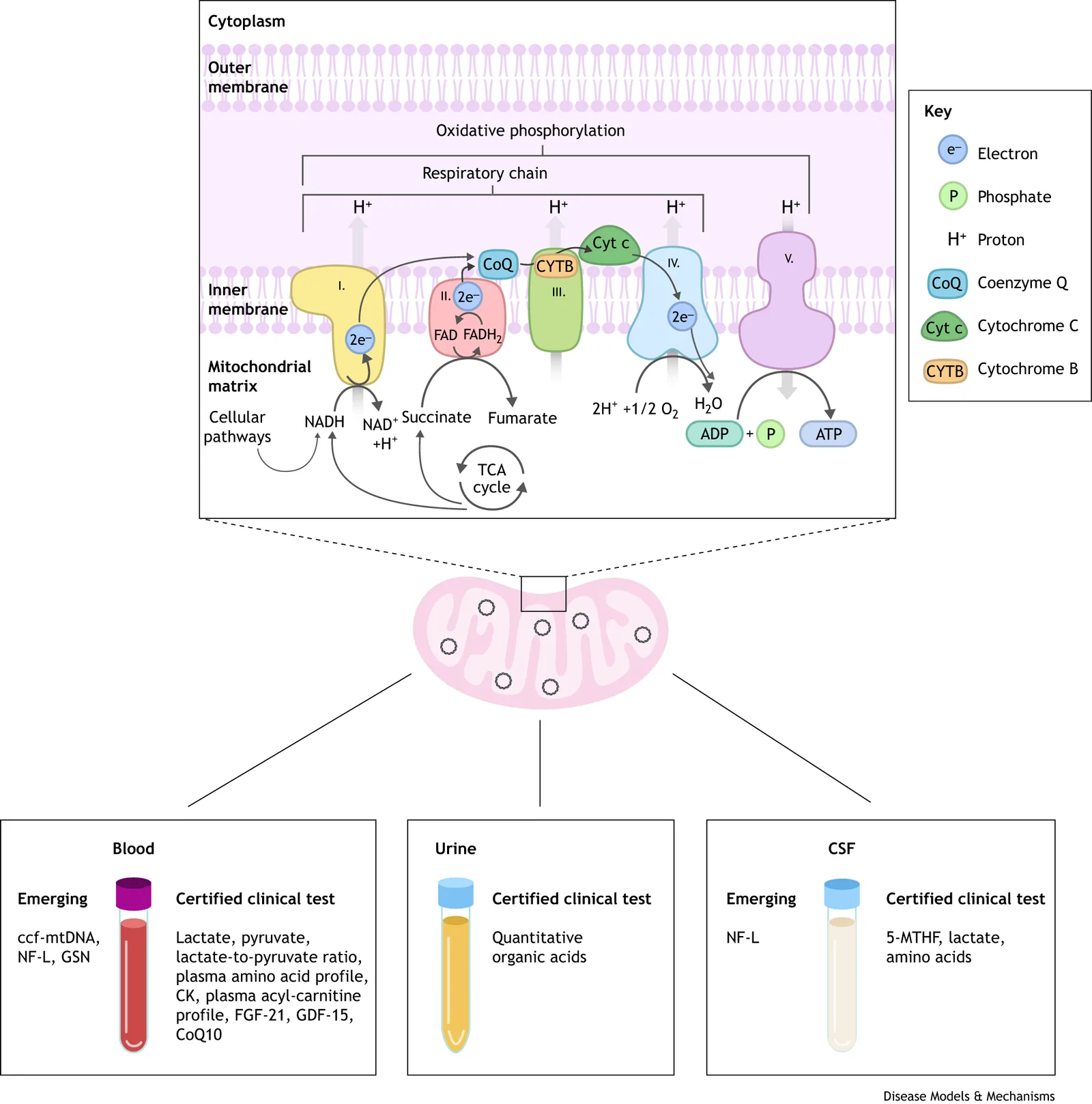
**Mitochondrial oxidative phosphorylation and laboratory analyses used in the diagnostic evaluation of PMD.** The upper panel shows the respiratory chain and ATP synthase within the inner mitochondrial membrane. Electrons derived from NADH and succinate enter through complexes I and II, respectively, and are transferred through CoQ, complex III, Cyt c and complex IV to oxygen. Proton translocation by complexes I, III and IV generates the gradient used by complex V to produce ATP. The lower panel summarizes analyses performed in blood, urine and CSF. Certified clinical tests include lactate, pyruvate, the lactate-to-pyruvate ratio, plasma amino acids, CK, the plasma acylcarnitine profile, FGF-21, GDF-15 and CoQ10 in blood; quantitative organic acids in urine; and 5-MTHF, lactate and amino acids in CSF. Circulating cell-free mtDNA, NF-L and gelsolin in blood and NF-L in CSF are shown separately as emerging analytes. These findings may support the diagnostic evaluation but should be interpreted together with the clinical presentation and molecular and other laboratory findings. ADP, adenosine diphosphate; ATP, adenosine triphosphate; ccf-mtDNA, circulating cell-free mtDNA; CK, creatine kinase; CoQ, coenzyme Q; CSF, cerebrospinal fluid; Cyt c, cytochrome C; CYTB, cytochrome B; e^−^, electron; FAD, flavin adenine dinucleotide; FADH, reduced flavin adenine dinucleotide; FGF-21, fibroblast growth factor 21; GDF-15, growth differentiation factor 15; GSN, gelsolin; H^+^, proton; H_2_O, water; mtDNA, mitochondrial DNA; 5-MTHF, 5-methyltetrahydrofolate; NADH, reduced nicotinamide adenine dinucleotide; NF-L, neurofilament light chain; P, phosphate; PMD, primary mitochondrial disease; TCA, tricarboxylic acid.

### Blood biomarkers biochemical testing

#### Plasma lactate and pyruvate

Elevated plasma lactate is a commonly measured biochemical parameter in the diagnosis of suspected PMD. However, its diagnostic utility in PMD is limited by poor (∼80%) sensitivity and specificity (Turton et al., 2022; Clarke et al., 2013). Although elevated plasma lactate can occur in PMD, it can be inconsistent, often transient and can be influenced by irrelevant factors, such as fasting state, intercurrent illness, recent exercise or exertion, and technical issues during sample collection (e.g. prolonged tourniquet application or delayed processing) (Clarke et al., 2013). Retrospective cohort studies have shown that although lactate can be elevated in some mitochondrial respiratory chain (RC) deficiencies, pyruvate and alanine levels might be more reliable, particularly when differentiating OXPHOS disorders (such as those caused by pathogenic variants in genes coding OXPHOS subunits or assembly factors) from pyruvate dehydrogenase complex deficiency (PDCD) (Clarke et al., 2013; Shayota, 2024). For greater diagnostic accuracy, lactate and pyruvate can be measured simultaneously to calculate the lactate-to-pyruvate ratio, which serves as a proxy for the cytosolic NADH/NAD^+^ redox state. A lactate-to-pyruvate ratio >20 in the setting of elevated lactate is common in RC dysfunction, whereas a normal ratio with parallel elevations of lactate and pyruvate is more commonly seen in PDCD (Table 1) (Varhaug et al., 2021; Debray et al., 2007). The underlying NADH/NAD^+^ imbalance, or ‘NADH-reductive stress’, in PMD drives pyruvate reduction to lactate and the accumulation of related metabolites (Sharma et al., 2021). When conducting these blood marker tests, strict sample handling is critical: blood should be drawn without a tourniquet where possible, patients should avoid recent meals or strenuous activity, and pyruvate must be stabilized immediately to prevent artifactual degradation (Turton et al., 2022). Overall, plasma lactate remains a useful diagnostic tool when assessed in combination with other biomarkers, such as pyruvate, alanine and the newer biomarkers discussed below, and with functional assays, to provide a more reliable picture of underlying mitochondrial dysfunction (Forny et al., 2021).

#### Plasma amino acid profile analysis

The quantitative analysis of plasma amino acid profiles is a valuable approach for diagnosing PMD, although no single amino acid pattern is pathognomonic. This analysis should ideally be performed on a morning fasting sample, as post-meal values can be misleading (Haas et al., 2008). A commonly recognized abnormality is hyperalaninemia. However, it is neither sensitive nor specific for PMD and should be interpreted together with other biochemical findings, particularly lactate, pyruvate and lactate-to-pyruvate ratio, because it may reflect persistent lactate and pyruvate accumulation through the transamination of pyruvate to alanine (Clarke et al., 2013). The diagnostic significance of hyperalaninemia increases when elevations are observed in both plasma and CSF, and when plasma is drawn under fasting conditions to exclude mild post-meal increases. Obtaining fasting samples may be challenging in clinical practice, particularly in pediatric or emergency situations. Evaluating alanine levels relative to essential amino acids helps differentiate true pathological hyperalaninemia from generalized, diet-induced amino acid fluctuations (Clarke et al., 2013). An alanine to lysine ratio ≥3 or an alanine to (phenylalanine+tyrosine) ratio ≥4 indicates disproportionate endogenous alanine synthesis and may provide additional biochemical support in the evaluation of PMD (Haas et al., 2008)

Some amino acid alterations can be suggestive of specific conditions. For example, low citrulline levels have been described in MT-ATP6-related disorders and can be detected on newborn screening, highlighting that certain amino acid changes might act as early diagnostic markers (Peretz et al., 2021; Tise et al., 2023). The finding is not limited to infancy. Adult maternal relatives with homoplasmic MT-ATP6 variants, ranging from asymptomatic to a neuropathy, ataxia, retinitis pigmentosa (NARP) phenotype (Table 1), show a similar biochemical pattern (Tise et al., 2023), and hypocitrullinemia is present in approximately two-thirds of patients with MT-ATP6 variants, more often with m.8993T>G (Li et al., 2025).

Elevated glycine is associated with lipoic acid synthetase (LIAS) deficiency (Table 1), a PMD affecting mitochondrial cofactor biosynthesis. Reduced LIAS activity impairs lipoate-dependent enzymes, including the glycine cleavage system, and causes variant non-ketotic hyperglycinemia, which resembles classical *GLDC*/*AMT*-related non-ketotic hyperglycinemia (NKH) (Baker et al., 2014). However, it can be distinguished from classical NKH by the accompanying lactic acidosis and combined 2-ketoacid dehydrogenase deficiency (Mayr et al., 2011).

The clinical syndrome mitochondrial encephalomyopathy, lactic acidosis and stroke-like episodes (MELAS) can present with low citrulline and arginine levels (Jong et al., 2021; Ohsawa et al., 2019). Alterations in proline and sarcosine have also been reported in mitochondrial dysfunction, although not in association with a specific PMD (Haas et al., 2008)

#### Creatine kinase

Creatine kinase (CK) is frequently measured in the evaluation of suspected PMD, particularly in cases with myopathic involvement, as an indicator of muscle breakdown (Conti et al., 2023). However, its diagnostic value is limited by poor sensitivity and specificity. Many patients with genetically confirmed PMD demonstrate normal or only mildly elevated CK, while elevations can also be observed in non-mitochondrial myopathies, inflammatory muscle diseases, or following exertion and dehydration (Ostojic, 2018). Despite these limitations, persistently elevated CK in the context of mitochondrial myopathies can provide supportive evidence, especially when interpreted alongside other biochemical or clinical findings (Shayota, 2024). Current consensus suggests that CK should not be relied upon as a stand-alone marker, but rather integrated into broader biomarker panels including lactate, alanine and emerging markers discussed below, such as FGF-21 and GDF-15 (Hubens et al., 2022)

#### GDF-15

GDF-15 (a stress-responsive cytokine) has emerged as a useful biomarker in some PMD subclasses and often outperforms traditional markers, such as lactate and CK. Elevated GDF-15 is particularly pronounced in patients with SLSMDs (MacMullen et al., 2025), and its levels differ across clinical syndromes. For example, patients with Kearns–Sayre syndrome (KSS), a mitochondrial DNA deletion disorder, have the highest GDF-15 levels, followed by those with the MT-tRNA^lys^ m.8344A>G variant causal of myoclonic epilepsy with ragged-red fibers (MERRF), followed next by patients with Pearson syndrome and related SLSMD disorders (Varhaug et al., 2021). GDF-15 can also be used to diagnose PMD in those with abnormal mtDNA content and/or pathogenic mutations. In a large cohort, a cut-off of 815 pg/ml yielded 76% sensitivity, 88% specificity and a negative predictive value of 97% for mtDNA-related disorders (Fernández et al., 2025). At this threshold, GDF-15 was elevated in 76% of mtDNA-PMD, compared to only 26% of nDNA-PMD and in 11% of non-mitochondrial controls, underscoring its value particularly for evaluating mitochondrial genome defects (Fernández et al., 2025).

#### FGF-21

FGF-21 is a stress-responsive hormone primarily produced in the liver and expressed in adipose tissue, skeletal muscle and pancreas, where it regulates glucose and lipid metabolism (Woo et al., 2013). Induction of FGF-21 expression occurs through mitochondrial stress pathways, and several studies have shown elevated circulating levels in patients with PMD (Suomalainen et al., 2011). The strongest associations are observed in mitochondrial myopathies, in which FGF-21 correlates with muscle involvement and disease severity (Lehtonen et al., 2021). However, FGF-21 levels do not consistently increase in patients with OXPHOS disorders or in the absence of prominent muscle involvement, highlighting its limitations as a universal biomarker (Turton et al., 2022). In addition, FGF-21 is elevated in common non-mitochondrial conditions, such as diabetes mellitus, obesity, metabolic syndrome, non-alcoholic fatty liver disease and chronic kidney disease (Woo et al., 2013). Furthermore, although FGF-21 is useful in diagnosing those with predominantly a myopathic phenotype, it is outperformed by GDF-15 in overall diagnostic sensitivity across genetically diverse PMD cohorts (Davis et al., 2016). This finding has since been reinforced by a meta-analysis confirming that although both FGF-21 and GDF-15 show acceptable sensitivity and high specificity, GDF-15 consistently demonstrated the highest diagnostic accuracy (Lin et al., 2020). Taken together, FGF-21 represents a valuable supportive biomarker of mitochondrial disease, especially in muscle-dominant phenotypes, but its clinical interpretation requires careful contextualization with other biomarkers and genetic data (Shayota, 2024).

#### Circulating cell-free mitochondrial DNA

Circulating cell-free mitochondrial DNA (ccf-mtDNA) is an emerging biochemical analyte released into the bloodstream during cellular stress or mitochondrial damage, where it can act as a damage-associated molecular pattern (DAMP) that triggers innate immune pathways and inflammatory signaling (Maresca et al., 2020; de Miranda et al., 2024). It has been investigated as a potential biomarker of PMD, because elevated plasma ccf-mtDNA levels have been reported in cohorts with mtDNA disorders, particularly in MELAS, in which ccf-mtDNA concentrations can rise further during stroke-like episodes (Trifunov et al., 2021). These studies suggest that evaluating ccf-mtDNA might complement the use of blood-based biomarkers, such as FGF21 and GDF-15, in certain PMD cohorts, especially when capturing acute events and inflammatory activity (Trifunov et al., 2021; Shayota, 2024). Despite its promise as a biomarker, ccf-mtDNA analysis remains restricted to research settings in specialized centers, with no standardized clinical assays currently available (Wen et al., 2025).

#### Gelsolin

Gelsolin is a conserved calcium-dependent actin-binding protein that regulates filament remodeling and has been investigated as a potential biomarker in PMD. Recent evidence indicates that its cytoskeletal functions extend to mitochondrial biology, influencing apoptotic signaling and cellular energy homeostasis (Illescas et al., 2021). Experimental models of OXPHOS deficiency have shown that the cytosolic isoform of gelsolin can be recruited to the mitochondrial outer membrane (mGSN), where it interacts with the voltage-dependent anion channel to modulate pore oligomerization and limit apoptosis, while the circulating plasma gelsolin (pGSN) levels are reduced (García-Bartolomé et al., 2020, 2017; Peñas et al., 2021). A higher mGSN:pGSN ratio has, therefore, been proposed as a cellular indicator of OXPHOS dysfunction. In clinical studies, decreased plasma gelsolin has been observed in patients with PMD, and its diagnostic performance appears strongest when combined with established biomarkers, such as FGF-21 and GDF-15, achieving high sensitivity and specificity (Peñas et al., 2021). Although these findings are promising, they require validation across diverse PMD cohorts before gelsolin can be established as a reliable PMD biomarker.

### Urine biochemical biomarkers

#### Urine organic acid quantitative analysis

Urine organic acid quantitative analysis remains a common first-line investigation in suspected PMD, although abnormalities are highly non-specific (Forny et al., 2021; Turton et al., 2022; Shatla et al., 2014). Elevations of metabolites [such as lactate, pyruvate, TCA cycle intermediates, ethylmalonic acid, methylmalonic acid and 3-methylglutaconic acid (3-MGA)] are frequently observed, yet overlap with findings seen in many other IEM disorders (Wortmann et al., 2015; Alston et al., 2021; Stenton and Prokisch, 2020). Importantly, beyond OXPHOS disorders, a broader group of IEM disorders with mitochondrial-localized enzyme deficiencies can mimic PMD. Branched-chain amino acid catabolic defects (such as isovaleric acidemia and maple syrup urine disease), and disorders of pyruvate metabolism (such as PDCD) and of TCA flux (such as SUCLA2/SUCLG1 deficiency, fumarase deficiency) produce biochemical profiles and clinical presentations that overlap with PMD (Turton et al., 2022). This is further complicated by the fact that branched-chain amino acid metabolism feeds into the TCA cycle and influences glycolytic flux, creating secondary increases in lactate and plasma amino acids that obscure interpretation of their altered levels in PMD urine samples (Vanweert et al., 2022). Similarly, increased 3-MGA excretion occurs in Barth syndrome, a disorder of cardiolipin remodeling, but is also found in a heterogeneous group of IEM disorders (Wortmann et al., 2013, 2012). Taken together, although urine organic acid quantitative profiles remain valuable for screening, no single metabolite pattern is diagnostic for PMD, and their careful integration with genetic and functional diagnostic analyses remains essential.

### Cerebrospinal fluid biomarker biochemistry

#### Lactate

The measurement of lactate in CSF is one of the oldest investigations of suspected PMD. Elevated CSF lactate can be observed even when plasma levels remain within the normal range, particularly in Leigh syndrome spectrum and other encephalopathic PMDs, including in patients with normal plasma lactate levels (Haas et al., 2007, 2008). Although both lactate and pyruvate can be measured in CSF, most consensus guidelines emphasize that the diagnostic value of the lactate-to-pyruvate ratio lies primarily in plasma, where it is used to help differentiate OXPHOS disorders from PDCD (Parikh et al., 2015, 2019). An elevated CSF lactate concentration can support impaired cerebral oxidative metabolism, but it is not specific to PMD and may also occur following seizures (Chatzikonstantinou et al., 2015).

#### Amino acids

CSF studies can provide additional supportive evidence for suspected cases of PMD (Turton et al., 2022). Elevated alanine in CSF is the most frequently detected abnormality, reflecting the chronic accumulation of lactate and pyruvate and transamination via alanine aminotransferase, which is consistent with impaired oxidative metabolism in the central nervous system (CNS) (Rahman, 2020). Increased glutamine has also been reported to result from altered nitrogen balance and secondary effects on neurotransmitter metabolism. (Bornstein et al., 2023). However, these abnormalities are not unique to PMD and overlap with other neurometabolic conditions, limiting their diagnostic value (Shayota, 2024). For this reason, CSF amino acid profiles are best considered as optional and, at best, complementary to plasma analyses, with meaningful interpretation requiring careful integration with clinical assessment and with blood lactate, pyruvate and genetic testing data (Boenzi and Diodato, 2018).

#### 5-Methyltetrahydrofolate

Deficiency of 5-methyltetrahydrofolate in CSF has been documented in several PMDs, including in KSS (Batllori et al., 2018). 5-Methyltetrahydrofolate transport across the choroid plexus is ATP dependent; thus, its reduced CSF levels might reflect impaired energy availability and also broader disruption of mitochondrial folate-mediated one-carbon metabolism. Mitochondrial dysfunction can impair the mitochondrial component of the one-carbon cycle, which normally generates formate and other one-carbon units required for cytosolic folate-dependent processes, such as 5-methyltetrahydrofolate recycling, methionine synthesis and homocysteine remethylation. Such perturbations can secondarily lower folate availability in the CNS and contribute to ‘secondary cerebral folate deficiency’ (Bao et al., 2016; Ducker and Rabinowitz, 2017; Hyland et al., 2010). In KSS, additional structural abnormalities of the choroid plexus may further restrict folate entry (Tanji et al., 2000). Oxidative stress can also contribute to reduced 5-methyltetrahydrofolate in the CSF, as reactive oxygen species can degrade folate species, with experimental studies linking this decrease to superoxide generation (Aylett et al., 2013). Accurate assessment of 5-methyltetrahydrofolate also requires a lumbar puncture under fasting conditions, sample protection from light and rapid freezing of CSF, with quantification by high-performance liquid chromatography (HPLC) or liquid chromatography–tandem mass spectrometry (LC-MS/MS). Overall, low 5-methyltetrahydrofolate levels in the CSF are a supportive biomarker of PMD, but they reflect both mitochondrial energy failure and secondary oxidative degradation, limiting specificity (Akiyama et al., 2015).

#### Neurofilament light chain

Neurofilament light chain (NF-L) is a cytoskeletal protein released into body fluids following axonal injury, and its measurement in CSF has proven informative in disorders such as Alzheimer’s disease and in traumatic brain injury (Coppens et al., 2023). Given the neurodegenerative nature of a substantial proportion of PMDs, NF-L has been considered a potential biomarker for both PMD diagnosis and progression (Sofou et al., 2019; Zheng et al., 2021). However, evidence on its use is limited to two studies conducted to date. One study reported elevated serum NF-L in MELAS, with peak levels occurring during acute stroke-like episodes and persistent elevation in remission (Zheng et al., 2021). The second study reported increased CSF NF-L in pediatric mitochondrial encephalopathy cases compared to controls, with higher concentrations linked to abnormal brain magnetic resonance imaging (MRI) findings and reduced survival (Sofou et al., 2019). These preliminary findings suggest that NF-L could serve as a marker of CNS involvement in PMD, but more studies are needed to establish its diagnostic and prognostic value.

## Noninvasive functional testing for PMD

### Cardiopulmonary exercise testing

Cardiopulmonary exercise testing (CPET) is used as a diagnostic tool for mitochondrial myopathy (MM), a subset of PMD, particularly when genetic testing yields inconclusive results about impaired mitochondrial function and when patients can perform this cardiopulmonary assessment (Parikh et al., 2015, 2019) (Fig. 4). Three key CPET parameters are indicative of PMD: a peak oxygen uptake (peak VO_2_) below 50% predicted; a peak ventilatory equivalent for oxygen (VE/VO_2_) above 50; and a peak cardiac output-to-oxygen uptake (ΔQ/ΔVO_2_) slope≥7 (Guazzi et al., 2012). When two or more of these thresholds are met, the likelihood of MM increases significantly (Bhatia et al., 2021). CPET also demonstrates that, at the anaerobic threshold, VO_2_ is reduced and ventilatory equivalents (VE/VO_2_ and VE/VCO_2_) are elevated, reflecting an early hyperventilatory response and cardiovascular impairment in MM (Bhatia et al., 2021). CPET results can also indicate underlying cardiac, pulmonary or general deconditioning as causes of exercise intolerance (Kabbadj et al., 2024).

**Fig. 4.**
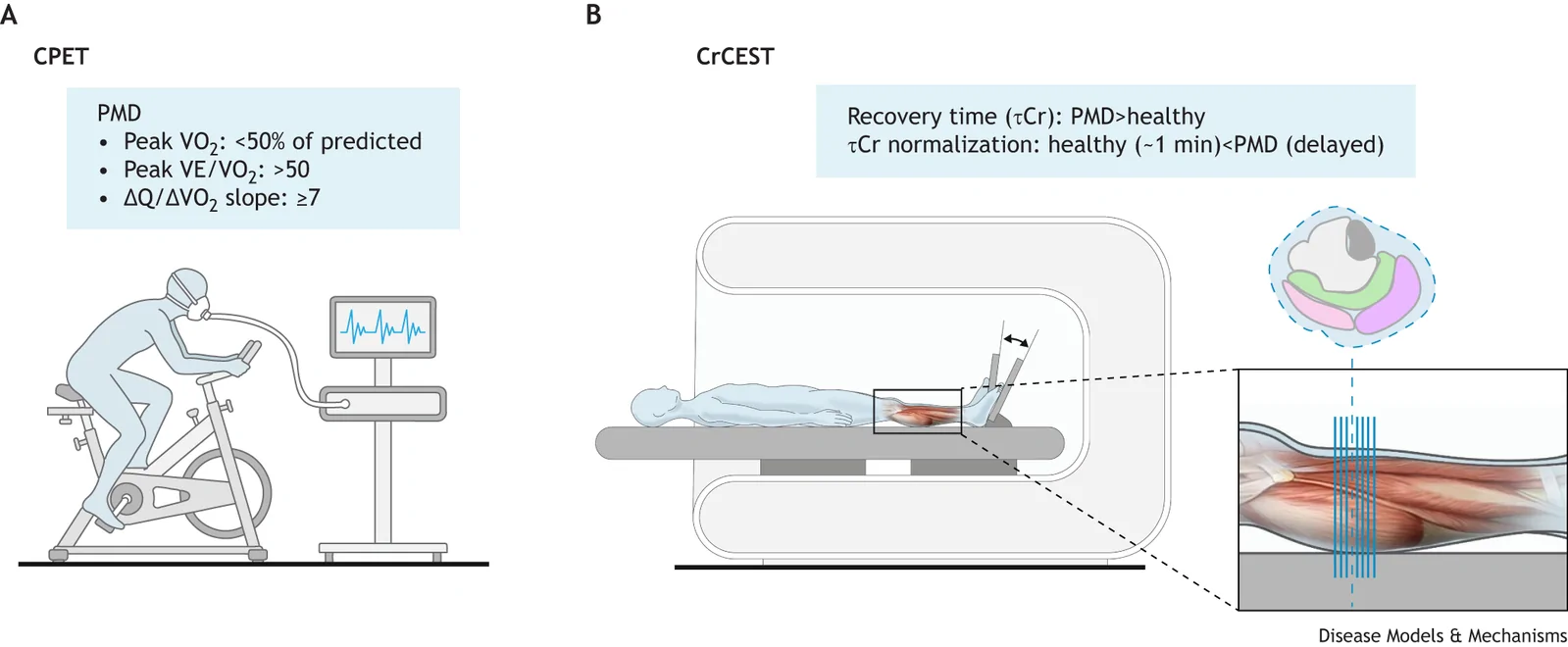
**Non-invasive functional assessment of mitochondrial oxidative phosphorylation.** (A) CPET measures the integrated cardiovascular, respiratory and skeletal muscle responses during graded exercise. In individuals able to complete maximal testing, a peak oxygen uptake (peak VO_2_) below 50% of the predicted value, a peak ventilatory equivalent for oxygen (VE/VO_2_) above 50, and a cardiac output-to-oxygen uptake slope (ΔQ/ΔVO_2_) ≥7 may raise suspicion for mitochondrial myopathy. (B) CrCEST MRI measures post-exercise changes in free creatine within individual skeletal muscles. Following plantar flexion exercise, serial images are acquired across selected calf muscles to determine the creatine recovery time constant (τCr). Recovery occurs within ∼1 min in healthy muscle but is delayed in primary mitochondrial disease, consistent with reduced muscle-specific OXPHOS capacity. CPET is used clinically in selected patients, whereas CrCEST MRI currently remains a research-based method. CPET, cardiopulmonary exercise testing; CrCEST, creatine chemical exchange saturation transfer; MRI, magnetic resonance imaging; PMD, primary mitochondrial disease; Q, cardiac output; τCr, creatine recovery time constant; VE/VO_2_, ventilatory equivalent for oxygen.

Although CPET offers objective and reproducible metrics for assessing exercise capacity and for quantifying muscle OXPHOS capacity, there are limitations to its widespread implementation in the diagnosis of PMD (Bhatia et al., 2021). Anecdotally, patients with PMD with significant ataxia, dystonia, cognitive delay, muscle weakness and/or fatigue are commonly unable to complete maximal CPET. Nevertheless, when combined with clinical diagnostic evaluation and genetic testing, CPET enables clinicians to noninvasively quantify whole-body muscle OXPHOS capacity during diagnostic evaluation. CPET parameters, such as peak or maximal oxygen consumption and the oxygen uptake efficiency slope, have also been shown to correlate directly with mtDNA heteroplasmy levels in PMD skeletal muscle, further underscoring the diagnostic value of CPET (Bhatia et al., 2021; Jeppesen et al., 2021).

#### Creatine chemical exchange saturation transfer

Creatine chemical exchange saturation transfer (CrCEST) MRI is a high-resolution and noninvasive approach for measuring muscle-specific OXPHOS. Although not yet in clinical use, it is being explored for diagnosing PMD (Fig. 4).

Clinicians currently lack the means to quantitatively measure, noninvasively and *in vivo*, mitochondrial energy production over time in individuals with suspected or confirmed PMD. CrCEST exploits the proton exchange that occurs between free creatine and water, enabling high-resolution mapping of creatine dynamics using standard proton (^1^H) MRI coils (Chakraborty et al., 2025; DeBrosse et al., 2016). CrCEST MRI is emerging as a valuable noninvasive imaging modality for assessing *in vivo* mitochondrial OXPHOS capacity in skeletal muscle metabolism (DeBrosse et al., 2016; Kogan et al., 2014). Recent studies have demonstrated the utility of CrCEST in quantifying the post-exercise recovery kinetics of creatine, specifically by measuring its recovery time constant in distinct skeletal muscles within the calf following a short (2 min) bout of plantar flexion exercise (Armbruster et al., 2024; Chakraborty et al., 2025; DeBrosse et al., 2016; Kogan et al., 2014). This noninvasive biomarker reflects muscle-group-specific mitochondrial OXPHOS capacity by capturing the biochemical resynthesis of phosphocreatine via the CK reaction, the recycling of which depends directly on OXPHOS capacity (Chance and Williams, 1955; Kogan et al., 2014). CrCEST MRI also provides muscle group-specific estimates of relative free creatine concentrations. Whereas phosphocreatine and free creatine concentrations normalize within ∼1-min post-exercise in unaffected individuals, this recovery is substantially delayed in patients with PMD, with the time to recovery reflecting the severity of OXPHOS capacity in that muscle (DeBrosse et al., 2016).

These findings highlight the potential utility of CrCEST for longitudinal studies and clinical trials, where repeated, noninvasive assessments of muscle bioenergetics are essential (Chakraborty et al., 2025; DeBrosse et al., 2016) but not previously possible. CrCEST MRI currently remains available only on a research basis (DeBrosse et al., 2016; Schur et al., 2022).

## Tisue-based assays

Tissue biopsy is widely considered the diagnostic gold standard for PMD, particularly as a second-line option when blood-based molecular investigations are inconclusive (Parikh et al., 2015). Biopsy-derived tissue enables mitochondrial function to be directly assessed using enzymatic activity assays, mtDNA genome sequence and content analysis, as well as by structural (histology, immunohistochemistry, electron microscopy) evaluations, providing essential data that complement and inform the clinical significance of genomic findings (DiMauro et al., 2013; Sciacco et al., 1994). Tissue biopsy samples are also increasingly being utilized as a substrate for multi-omics analyses in research and clinical diagnostic pipelines. In cases in which VUSs are identified on molecular testing, integrated molecular and functional evaluation of affected tissue remains crucial for confirming an accurate PMD diagnosis (Fig. 2) (Alston et al., 2021). This evolving role of tissue biopsy reflects the broader transition of PMD diagnostics from a biopsy-first to a genomics-first paradigm, in which unbiased NGS strategies performed on blood now achieve molecular diagnosis in many patients without an initial need for tissue biopsy or biopsy-directed candidate gene sequencing (Wortmann et al., 2015)

Clinicians use a variety of tissue sources for biopsies when evaluating PMD. Diagnostic consensus guidelines emphasize that the tissue most profoundly affected by the specific disease process, such as the liver in hepatopathy, cardiac muscle in cardiomyopathy, or skeletal muscle for myopathy, should ideally be targeted for functional assays (Fig. 2) (Haas et al., 2008; Parikh et al., 2015). However, skeletal muscle is the preferred tissue for the diagnostic evaluation of PMD for a range of reasons. These include its high metabolic workload, postmitotic status, accessibility, vulnerability to impairments in OXPHOS capacity, involvement in myopathy and in isolated and multisystemic phenotypes, and the standardization of laboratory assessments of muscle tissue relative to healthy controls (Alston et al., 2021). Muscle tissue also often exhibits higher levels of mtDNA heteroplasmy and mosaicism in biochemical deficiency relative to those present or detectable in cultured cells. However, muscle biopsies are highly invasive procedures that typically require anesthesia, carrying surgical risks, and are not easy to repeat (Driessen et al., 2007; Parikh et al., 2015). Skin biopsy offers a less invasive alternative to muscle biopsy and enables the culture of primary fibroblast cell lines for downstream functional testing (Gill et al., 2023; Oglesbee et al., 2006), including electron transport chain (ETC) enzymology, blue native polyacrylamide gel electrophoresis (BN-PAGE) and respirometry analyses, as discussed in the following sections.

### Histology

Histology is routinely performed on biopsied material to inform the clinical evaluation of PMD. Globally, specialized diagnostic centers and a selected number of reference laboratories offer clinical histological analyses of muscle. Although histologic abnormalities are not always overt in PMD, particularly in children, myopathic changes may be seen on Hematoxylin and Eosin staining, including variation in fiber size and shape, increased numbers of internally located nuclei, and changes suggestive of denervation or mitochondrial proliferation (Petruzzella et al., 1994). No universal histological muscle feature is associated with PMD. However, a hallmark feature that does raise suspicion of PMD is the presence of ragged-red fibers (RRFs) on Gomori trichrome staining, characterized by subsarcolemmal mitochondrial accumulation and a disrupted fiber border. Although RRFs are suggestive of PMD, they are not specific and can also be observed in aging muscle and in other neuromuscular disorders (Vogel, 2001). Similar to RRFs, ragged-blue fibers indicative of mitochondrial proliferation may be observed on nicotinamide adenine dinucleotide tetrazolium reductase (NADH) and/or succinate dehydrogenase (SDH) staining. Another histological finding of diagnostic value is the presence of cytochrome c oxidase (COX)-negative/SDH-positive fibers, seen in individual or combined COX/SDH histochemical staining. Three subunits of complex IV are mtDNA encoded, whereas complex II is encoded entirely by the nuclear genome. As a result, a transcription or translation defect that impairs OXPHOS reduces complex IV activity while complex II activity is preserved (Rocha et al., 2015; Taylor and Turnbull, 2005; Phadke, 2017). Additional histochemical abnormalities that may be observed in PMD include lipid accumulation within fibers and a predominance of type 1 fibers (Fig. 5).

**Fig. 5.**
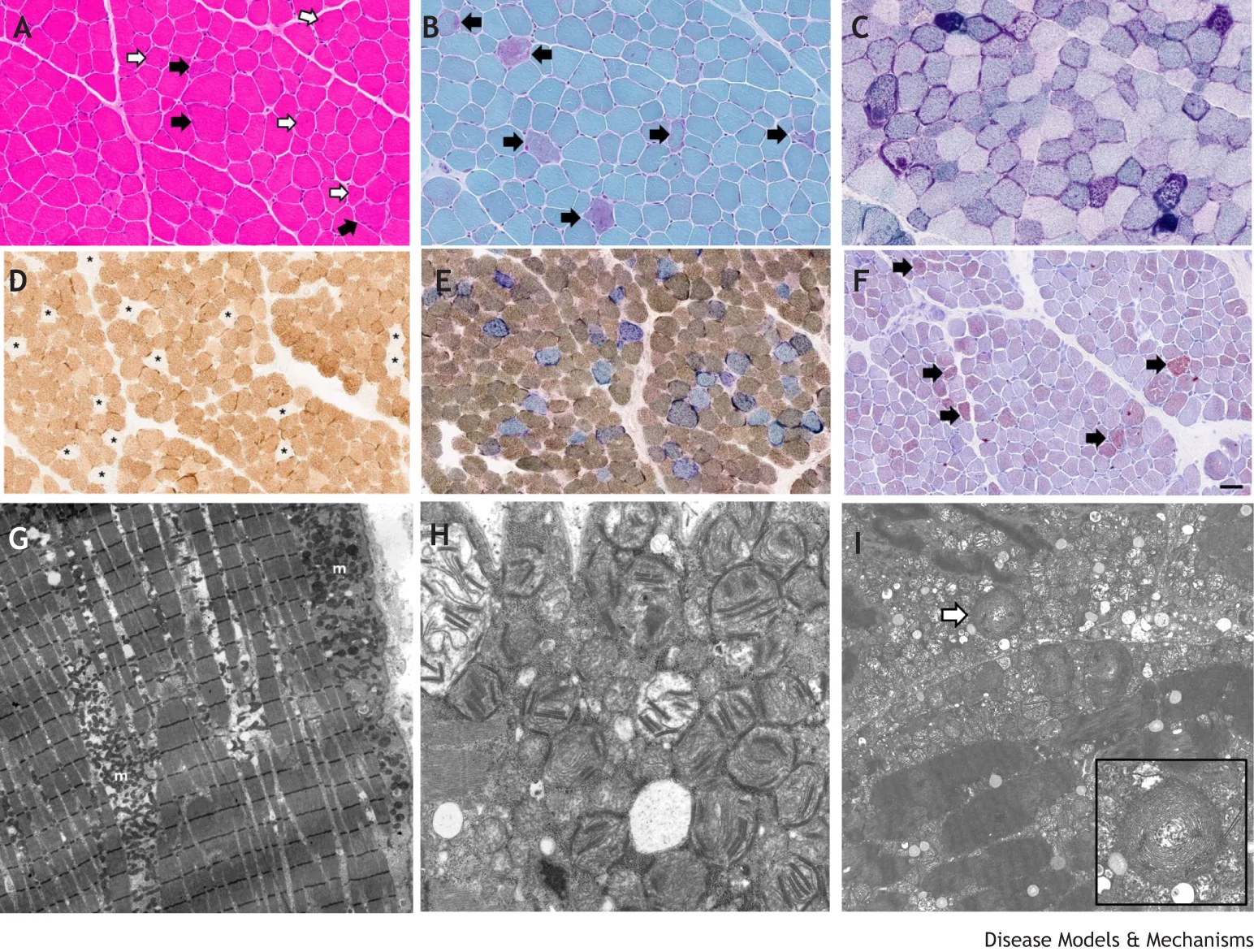
**Representative skeletal muscle histopathological and ultrastructural findings in primary mitochondrial disease.** (A) Hematoxylin and Eosin-stained section showing variation in muscle fiber size and shape, fibers with internally located nuclei (white arrows) and fibers with mitochondrial proliferation (black arrows), characterized by increased basophilic staining, particularly in the subsarcolemmal region. (B) Modified Gomori trichrome-stained section showing ragged-red fibers (black arrows). (C) NADH-TR staining showing ragged-blue fibers with increased subsarcolemmal and intermyofibrillar staining. (D) COX staining showing numerous COX-negative fibers (asterisks). (E) Combined SDH/COX staining showing COX-negative/SDH-positive fibers in blue. (F) Oil Red O staining showing lipid accumulation within muscle fibers (black arrows). (G) Electron micrograph showing mitochondrial proliferation in the subsarcolemmal region and within the muscle fiber (m). (H) Electron micrograph showing enlarged mitochondria with abnormal cristae and numerous crystalline inclusions. (I) Electron micrograph showing mitochondrial proliferation, including a giant mitochondrion with concentric cristae (white arrow), shown at higher magnification in the inset. Original magnifications: A–F, ×400; G, ×3500; H, ×15,000; I, ×5000; I, inset, ×10,000. Scale bar: 50 μm. COX, cytochrome c oxidase; NADH-TR, nicotinamide adenine dinucleotide–tetrazolium reductase; SDH, succinate dehydrogenase.

To improve diagnostic resolution, quadruple immunofluorescence-based assays have also been developed to quantify RC subunits, particularly in complexes I and IV, alongside mitochondrial mass markers, such as the outer mitochondrial membrane protein, voltage-dependent anion channel (also known as mitochondrial porin). These multiplexed methods, performed on a single cryosection, enable fiber-level quantification of OXPHOS deficiency and enable mosaic patterns to be detected that might be missed by bulk enzymatic assays (Rocha et al., 2015).

### Electron microscopy

Electron microscopy provides tissue ultrastructure information that complements molecular and histochemical assays in the evaluation of PMD (Fig. 5) (Vincent et al., 2016). PMD is associated with several abnormalities detectable by electron microscopy, including proliferation of mitochondria, giant mitochondria, paracrystalline inclusions (regularly arranged crystalline protein arrays within the mitochondrial intermembrane space) (Hammersen et al., 1980), linearized cristae and abnormal angular arrangements (sharply bent cristae profiles forming acute angles instead of the typical lamellar morphology) (Vincent et al., 2016). Sub-mitochondrial compartmentalization, in which multiple isolated matrix spaces exist within a single mitochondrion, has also been described in biopsies of patients with PMD and in PMD model organisms (Zick et al., 2009). Electron microscopy studies have also reported the presence of concentric ‘onion-like’ cristae, a phenomenon also reproduced in model systems, following the disruption of mitochondrial contact site and cristae organizing system (MICOS) complex proteins or ATP-synthase dimerization (Vincent et al., 2016). In patients with MICOS13 (QIL1) deficiency, mitochondria in cultured fibroblasts and in skeletal muscle instead display swirl-like inner membranes with loss of cristae junctions (Guarani et al., 2016). Hyperbranched and donut-shaped mitochondria, reflecting altered fission–fusion dynamics under stress conditions, have also been reported in patient biopsies and in experimental systems (Leduc-Gaudet et al., 2015; Vincent et al., 2016).

Nevertheless, electron microscopy requires specialized equipment and expertise, and is therefore not part of the standard diagnostic workup, although its findings can strengthen a suspected diagnosis of PMD.

### Mitochondrial respiratory chain enzymatic activity

Although NGS has become the first-line diagnostic approach, biochemical assessment of mitochondrial ETC enzyme activity remains the gold standard for functional confirmation in patients with suspected PMD, particularly when molecular genetic testing identifies a VUS (Alston et al., 2021; Wortmann et al., 2017; McCormick et al., 2018; Shen et al., 2020). These spectrophotometric assays evaluate the enzymatic activities of complexes I–IV in mitochondria isolated from skeletal muscle, cultured primary fibroblast cell lines, liver, kidney and, when available, cardiac tissue (Muraresku et al., 2018). Results are typically normalized to internal mitochondrial mass markers such as citrate synthase (CS) (Kirby et al., 2007).

An accepted diagnostic threshold defines major biochemical criteria for RC deficiency as enzymatic activity of any complex falling below 20% of the control mean in a tissue, below 30% in a cell line, or below 30% for the same complex in two or more tissues. Activities of 20–30% in a tissue, 30–40% in a cell line, or 30–40% for the same complex in two or more tissues constitute minor biochemical criteria (Bernier et al., 2002). A greater likelihood of PMD is indicated when complex-specific enzyme activity corrected levels fall below 20% of the control mean (Bernier et al., 2002). Although fibroblast cell line testing is less invasive and easier to repeat, skeletal muscle remains the preferred tissue for reasons explained in the previous section (Parikh et al., 2015, 2019; Taylor and Turnbull, 2005; Wortmann et al., 2015). Importantly, skeletal muscle may provide diagnostic information even in patients without overt myopathic features; however, a normal muscle result does not exclude PMD (Phadke, 2017).

Discrepancies across tissue types are also well recognized and can arise due to tissue-specific expression patterns of specific causal gene disorders or to compensatory *in vitro* metabolic changes (Oglesbee et al., 2006; Wen et al., 2025). As a clear example, in patients with a confirmed genetic diagnosis and a clear complex I deficiency in muscle, the deficiency is not always demonstrable in fibroblasts (van den Heuvel et al., 2004).

Technical and physiological variables, such as disuse atrophy, malnutrition or freeze–thaw cycles, can also reduce CS levels, leading to false-negative interpretations (Alston et al., 2021). Despite these challenges, ETC enzymatic analysis remains a valuable functional readout when interpreted alongside clinical, molecular and histological assessment. It continues to be the gold standard in specialized diagnostic Mitochondrial Medicine Centers worldwide (Gill et al., 2023), in which ETC enzyme activity assessment is offered for several tissues (including muscle, fibroblasts, liver and heart). Although ETC enzyme activity analysis conducted on buccal samples (relating to, involving, or located near the cheek or the inside of the mouth) is clinically available in the USA, it appears to be neither highly sensitive nor specific for PMD, nor reliably consistent relative to ETC enzyme activities quantified in other tissues from the same individual (Goldenthal et al., 2012; Muraresku et al., 2018).

### Electrophoretic analyses of mitochondrial complex assembly

BN-PAGE enables mitochondrial OXPHOS complexes to be separated and visualized under non-denaturing conditions (Jha et al., 2016; Wittig et al., 2006). This method maintains the complexes in their fully assembled state and so provides information on the enzymatic activity and structural integrity and stability of individual complexes and supercomplexes (Singh and Duchen, 2022). Mitochondrial proteins are typically isolated from patient-derived tissues, such as skeletal muscle or cultured skin fibroblasts, using mild detergents to preserve native protein–protein interactions. Under non-denaturing electrophoretic conditions, OXPHOS complexes migrate according to their molecular mass and can be visualized by Coomassie Blue staining, in-gel activity assays, or immunoblotting with subunit-specific antibodies (Van Coster et al., 2001). These visualization methods enable the abundance, assembly status and catalytic activity of all four RC complexes to be evaluated. BN-PAGE, thus, enables detection of catalytic, structural and assembly defects, as well as OXPHOS complex instability (Jha et al., 2016). Furthermore, comprehensive functional evaluation using BN-PAGE can help overcome well-recognized tissue-specific diagnostic discrepancies, which were mentioned in the ‘Respirometry’ section. As recently reported in a large-scale preprint study, although RC enzymology alone yields a limited diagnostic sensitivity and specificity in primary fibroblasts, combining it with BN-PAGE and complex assembly profiling significantly increases overall diagnostic sensitivity (Van Hove et al., 2026 preprint).

Following separation of OXPHOS complexes by BN-PAGE, complex I western blot analysis can be used to detect aberrant complex I assembly due to an isolated complex I defect or to combined OXPHOS deficiencies (Friederich et al., 2021), including reductions in the holocomplex or pathologic accumulations of complex I assembly intermediates that may not manifest as frank RC deficiencies or as BN-PAGE abnormalities. It supplements mitochondrial ETC enzymatic analysis and BN-PAGE, and is currently clinically available at one diagnostic laboratory in the USA (Table 2) (Friederich et al., 2017).

### Respirometry

Respirometry provides a functional readout of mitochondrial OXPHOS by measuring integrated oxygen consumption capacity in intact or permeabilized cells, tissues or isolated mitochondria (Wenchich et al., 2003). Classical polarographic assays measure the decline in dissolved oxygen within a sealed chamber as the sample respires. A Clark-type oxygen electrode records oxygen concentration while substrates and inhibitors are added sequentially to assess individual complexes, for example, glutamate/malate for complex I, succinate for complex II, and N,N,N′,N′-tetramethyl-p-phenylenediamine/ascorbate for complex IV-linked respiration. Coupled (state 3, ADP-stimulated) and resting (state 4, ADP-depleted) respiration can be compared to calculate the respiratory control ratio, a measure of OXPHOS coupling efficiency (Turton et al., 2022).

More recent high-resolution platforms, such as the Oxygraph-2k (Oroboros Instruments) and Seahorse XF pro (Agilent Technologies) analyzers, enable real-time assessment of oxygen consumption. For example, Seahorse analysis is used to assess extracellular acidification, which can be indicative of lactate production and pH, thereby informing functional evaluations of mitochondrial and glycolytic activity (Gill et al., 2023). These platforms can also be combined with complex-specific inhibitors, enabling the measurement of ATP-linked respiration, maximal capacity and spare capacity (Djafarzadeh and Jakob, 2017). Although these techniques are highly informative for mechanistic studies and biomarker validation, they remain largely confined to research settings rather than routine diagnostics. Polarographic testing by Oxygraph-2K analysis in cultured patient fibroblast cell lines is currently available in one clinical diagnostic laboratory in the USA (Table 2).

### Coenzyme Q10 determination

Primary coenzyme Q10 (CoQ10) deficiency is a rare but potentially treatable PMD caused by pathogenic variants in genes that encode CoQ10 biosynthetic proteins. Its accurate diagnosis guides appropriate treatment, prognostic assessment and genetic counseling (Hirano et al., 2006). Affected genes include *COQ2*, *COQ4*, *COQ5*, *COQ6*, *COQ7*, *COQ8A* (*ADCK3*), *PDSS1*, *PDSS2* and *COQ9* (Mantle et al., 2023; Salviati et al., 1993). CoQ10 deficiency is indicated in patients presenting with cerebellar ataxia, steroid-resistant nephrotic syndrome, infantile multisystem disease, or isolated or early-onset myopathy (Emmanuele et al., 2012; Montero et al., 2008). Given the wide pathogenic variant spectrum and variability of CoQ10 deficiency, exome sequencing or genome sequencing are now the preferred initial diagnostic tests for it (Yu et al., 2019). If VUSs are identified in CoQ10-related genes, or if clinical presentation strongly suggests CoQ10 deficiency but sequencing results are non-diagnostic, muscle biopsy can be performed to quantify CoQ10 via HPLC or LC-MS/MS to confirm the biochemical deficit (Mantle et al., 2023; Montero et al., 2008). Muscle tissue often yields more reliable CoQ10 levels relative to fibroblasts because of muscle's higher mitochondrial content and higher expression levels of CoQ10 biosynthetic enzymes (Hargreaves et al., 2020). Complex II+III activity can also be used to assess CoQ10 status in muscle and in fibroblasts. This assay depends on endogenous CoQ10 to link succinate oxidation to cytochrome c reduction, so a deficiency lowers complex II+III activity while complex II and complex III activities measured separately remain normal (Rahman, 2020). Adding a short chain analog such as CoQ1 to the assay restores activity, which supports the diagnosis. In fibroblasts, the reduction is often partial rather than clear cut, so this assay is used together with genetic testing rather than on its own (Shalata et al., 2019; Schon et al., 2021). When CoQ10 deficiency has been confirmed, oral CoQ10 supplementation can improve outcomes before irreversible damage occurs, although this approach is not universally successful owing to poor tissue bioavailability (Xie et al., 2021). Newer routes of CoQ10 delivery or formulation with greater bioavailability remain under investigation. Muscle testing for CoQ10 deficiency is available in at least one clinical diagnostic laboratory in the USA (Table 2).

Although the diagnostic landscape for PMD has largely shifted to a genomics-first approach, tissue-based assays remain a critical component of the diagnostic algorithm. Their diagnostic value depends strongly on the tissue examined. If it is accessible and clinically feasible, testing should be performed in the most severely affected tissue. As discussed earlier, this is particularly important for VUSs and variants in candidate genes, for which demonstration of a biochemical or functional defect in affected tissue can provide evidence of pathogenicity. Omics analyses may be used when targeted tissue studies are inconclusive or performed in parallel with conventional tissue-based assays to provide additional evidence and characterize the downstream effects of a candidate variant.

## Omics technologies in diagnosis of PMD

Although genomic analysis is often the first clinical step in the diagnostic journey, it might not provide a definitive causal explanation. To address this gap, multi-omics technologies have emerged to explore the disease process across multiple biological dimensions, spanning RNA (transcriptomics), proteins (proteomics) and metabolites (metabolomics) (Fig. 2). The use of multi-omics in the diagnostic process has been shown to facilitate the diagnosis of rare diseases, including PMD. Transcriptomic profiling, also known as RNA sequencing, is reported to aid in the diagnosis of PMD in ∼10% of cases, relative to WES analysis alone, by identifying altered gene expression, splicing defects and/or mono-allelic expression (Kremer et al., 2017). Proteomics is another valuable tool that can help to diagnose unsolved PMD. Studies show that proteomics results are consistent with the known genetic etiology of 83% of analyzed patients with PMD (Hock et al., 2025). Proteomics also facilitates the detection of multi-protein, complex-wide effects, such as global reductions in assembled mitochondrial complexes or mitoribosome subunits, and has been instrumental in validating variant pathogenicity in newly discovered mitochondrial disease genes, including *TIMMDC1* (Kremer et al., 2017), *NDUFAF8* (Alston et al., 2020) and *MRPS34* (Lake et al., 2017)*.* When proteomics is combined with complexome profiling, or with RNA sequencing and ETC enzymatic analyses, it provides powerful orthogonal evidence to support variant interpretation (Argelaguet et al., 2018). Untargeted proteomics can also capture functional readouts of NADH-reductive stress, identifying novel analytes such as N-lactoyl-amino acids and β-hydroxy-acylcarnitines, thereby extending the utility of proteomics beyond protein abundance to mechanistic insights into redox imbalance and metabolic stress in PMD (Sharma et al., 2021).

Metabolomics is also emerging as a powerful approach for diagnosing PMD, enabling the detection of distinct biochemical patterns that complement genomic data. Untargeted metabolomics profiling has uncovered potential biomarkers in patients with PMD, such as aberrant acylcarnitine species, amino acid derangements and lipid abnormalities, which correlate with disease severity and phenotypic variation (Gucek and Sack, 2021; Sharma et al., 2021; Gervasoni et al., 2024). In addition to established biochemical patterns, untargeted platforms can also capture signatures of the underlying cellular pathophysiology. For example, profiling of patients with MELAS with the m.3243A>G variant in *MT-TL1* identified new classes of circulating metabolites, such as N-lactoyl-amino acids, which reflect intracellular NADH-reductive stress (Sharma et al., 2021; Gervasoni et al., 2024). Given the wide phenotypic variability associated with m.3243A>G, even among patients with similar tissue heteroplasmy levels (Grady et al., 2018; Pickett et al., 2018), identifying related metabolic signatures via metabolomics can provide additional information about the biochemical effects of the variant.

Thus, metabolomics may provide another useful layer in the diagnostic pipeline for PMD, especially in cases in which genomic results alone do not provide definitive answers. Omics analyses can provide functional evidence when genomic testing is inconclusive, identifies a VUS, or reveals a potentially relevant variant in a candidate gene without an established Online Mendelian Inheritance in Man (OMIM) disease association. Transcriptomics can detect altered gene expression and splicing, proteomics can identify changes in mitochondrial protein abundance and complex assembly, and metabolomics can reveal downstream biochemical and redox abnormalities. Used individually or together with genomic, biochemical and tissue-based findings, these approaches can support variant interpretation and help define the consequences of genetic findings. However, their clinical implementation remains limited by sample and tissue requirements, access to specialized platforms, the availability of appropriate reference data and the need for further validation.

## Clinical application and challenges

The clinical translation of advances in PMD diagnosis is complex, primarily owing to the extensive genetic and phenotypic heterogeneity of these disorders. This section explores the challenges of genotype–phenotype correlation, disparities in diagnostic accessibility, ethical considerations in genomic medicine, and implications of these challenges for developing effective therapeutic strategies for the diverse forms of PMD.

### Genotype–phenotype correlations: predicting disease progression and outcomes

Translating genetic findings into accurate prognostic and treatment plans remains a major clinical challenge owing to highly unpredictable genotype–phenotype correlations, as discussed earlier. For example, the same pathogenic variant in the *POLG* gene might result in Alpers syndrome in one individual and in progressive external ophthalmoplegia (PEO) in another (Rahman and Copeland, 2019). Phenotypic evolution over time has also been observed in SLSMD syndromes, whereby patients initially presenting with Pearson syndrome might later meet the criteria for KSS and, ultimately, develop chronic progressive external ophthalmoplegia (CPEO), reflecting a continuum of increasing multi-system functional impairment over time (Alves et al., 2025; Ganetzky et al., 2025). To improve diagnostic accuracy and to inform optimal clinical trial design, recent efforts have focused on integrating multi-omics with multi-modal data. Clinical electronic medical record and research data integration and visualization platforms such as MMFP-Tableau incorporate genomic, biochemical and patient-reported outcome measures to generate a more granular understanding of disease subtypes, features and progression patterns (George-Sankoh et al., 2024; MacMullen et al., 2024). Despite these bioinformatics advances, many genomic variants remain classified as VUSs, limiting their clinical utility. Functional validation using patient-derived fibroblasts, induced pluripotent stem cells (iPSCs) or preclinical animal models has helped to address this challenge by enabling variant-specific phenotyping, but these methods are resource intensive, technically complex and largely confined to specialized research centers (Chen and Guan, 2023; McKnight et al., 2021; Falk, 2021).

Improving genotype–phenotype correlation ultimately depends on establishing effective global harmonization of diagnostic standards, broader data sharing and the expansion of well-curated longitudinal natural history studies (Keshavan and Rahman, 2018; Labory et al., 2020). The development of patient registries by mitochondrial disease advocacy groups and by consensus data element initiatives supports consistent data collection and facilitates the standardization of outcome measures (Karaa et al., 2018). Recent efforts have emphasized the importance of selecting standardized and clinically meaningful outcome measures that can be used consistently across both interventional trials and natural history studies in PMD (Goldstein and Rahman, 2021; Karaa et al., 2018; Flickinger et al., 2021; McCormick et al., 2025).

### Ethical considerations and access to diagnostics

Access to comprehensive mitochondrial disease diagnostics remains uneven across health systems. Even in high-income countries, health insurance policies often continue to cover WES more readily than WGS, contributing to delays in clinical diagnostic testing or to denials for some patients (Phillips et al., 2022). Multi-omics strategies also present accessibility challenges. The successful implementation of these advanced technologies requires substantial computational and interpretative infrastructure, which remains unavailable in many regions (Schon et al., 2021). In low- and middle-income countries, scarcity of sequencing infrastructure, trained personnel and bioinformatics capacity further limits access to genomic testing and interpretation (Bazalar-Montoya et al., 2024; Meldau et al., 2025). Although multi-omics analyses can improve our ability to diagnose challenging PMD cases, their effective translation into the clinic will require standardized pre-analytic processes, robust analytic pipelines and computing capacity typically concentrated in specialist centers (Kremer et al., 2017). The effective integration of systems biology frameworks that truly synthesize genomic, transcriptomic, proteomic and metabolomic data remains underutilized, despite their potential to democratize diagnostics via open-access platforms and standardized analytical pipelines (Labory et al., 2020; Schon et al., 2021; Stenton and Prokisch, 2020).

Ethical considerations remain central to many issues in diagnostic genomic testing. Incidental findings, particularly those involving adult-onset conditions identified in pediatric patients, can complicate the informed consent process (Karaa et al., 2018; Ormond et al., 2023; Eichinger et al., 2021; Nolan et al., 2024). mtDNA inheritance adds further complexity, raising considerations for maternal transmission and potential implications for family members (Miller et al., 2023; MacMullen et al., 2024; McCormick et al., 2020; Castelluccio et al., 2025). Institutional policies on recontacting patients when variants are reclassified vary widely. As reclassification becomes more frequent with improved data curation and interpretation, the need for transparent frameworks and ethical stewardship is increasingly urgent (Thummala et al., 2024; Goh et al., 2024).

Efforts to improve resource access to aid PMD diagnosis must prioritize international coordination, funding mechanisms for rare disease diagnostics, publicly available variant databases and greater access to genetic counseling. The integration of diagnostic pathways with therapeutic pipelines, as illustrated by advances in precision genetic editing or replacement therapies, as well as the development of hypoxia-modulating drug therapies for mitochondrial disease, might provide further justification for system-wide investment (Falk, 2025).

In conclusion, although technological advances have dramatically expanded the diagnostic capabilities and technological potential to improve our understanding of the complex causes of PMD, persistent ethical, structural and interpretational challenges demand thoughtful consideration. Global collaborative strategies are required to close the diagnostic gap and to ensure that every individual with suspected mitochondrial disease receives equitable and timely diagnosis-informed care.

## Future directions

As PMDs continue to challenge conventional diagnostic approaches, future work must prioritize the functional contextualization of genetic data (Gropman et al., 2024). Genomic sequencing, although transformative, often continues to leave unanswered questions in cases involving non-coding variants, VUSs or atypical clinical phenotypes (Davis et al., 2022; Mavraki et al., 2023). Increasing evidence supports the integration of multi-omics platforms – particularly transcriptomics, proteomics and metabolomics – as a way to bridge this gap by capturing downstream biological effects that clarify variant pathogenicity (Hock et al., 2025). Moving forward, diagnostic pipelines should evolve from static sequence analysis toward dynamic, cell-contextual diagnostics that reflect true disease biology (Wen et al., 2025). Multi-omics approaches should also revisit classical biochemical assays, such as urine organic acid analysis, using unbiased metabolomics pipelines, to determine whether complex metabolite signatures or ratios, not only single analytes, might improve diagnostic specificity for PMD (Wortmann et al., 2015). In this way, a more systematic approach to investigating PMD biomarkers will improve our understanding of known pathophysiology, which is particularly important given the overlap in biochemical patterns between PMD, other IEMs and secondary mitochondrial dysfunction. By combining genomics with transcriptomic, proteomic, metabolomic and functional datasets, it may be possible to define composite signatures that capture disease-specific pathways and to discriminate PMD from related conditions. Achieving this vision will require standardized protocols, validation across large genetically confirmed PMD cohorts, and longitudinal studies that track biomarker dynamics over time, ultimately transforming non-specific assays into components of precision diagnostic pipelines.

One key future imperative in the new age of artificial intelligence and machine learning involves continuing to build computational frameworks that consider and successfully accommodate the specific unique challenges of PMDs (Falk et al., 2015; Shen et al., 2024). Unlike common diseases, PMDs often involve private variants, small sample sizes and heterogeneous tissue expression (Kuo et al., 2025). Therefore, algorithms capable of managing sparse and noisy data, while detecting outlier molecular signals, are needed (Argelaguet et al., 2018; Wang et al., 2023). Future work should also prioritize the establishment of comprehensive healthy reference atlases – including tissue-specific transcriptomic and proteomic maps – that provide a normative baseline against which disease-specific deviations can be more accurately interpreted.

Equally important is the expansion of patient-derived experimental models, particularly primary fibroblast cell lines, iPSCs and organoids, which offer scalable, patient-specific platforms to test hypotheses generated by omics data (Chen and Guan, 2023). These models enable mitochondrial dysfunction to be investigated at multiple levels, spanning structural defects in OXPHOS complexes, altered metabolic flux and cellular stress responses under disease-relevant conditions (Heiduschka and Prigione, 2025; Hung et al., 2016). Beyond mechanistic insights, fibroblasts and iPSC-derived platforms have increasing potential for personalized drug screening, therapeutic target validation and even preclinical trial stratification (Hung et al., 2016; Liang, 2024; Falk, 2021).

Finally, the translation of integrative diagnostic strategies into routine clinical practice will require careful consideration of both logistical and ethical challenges (Stark et al., 2019). Essential steps will continue to include standardization of tissue collection and processing protocols to ensure reproducibility across centers, establishment of reimbursement pathways that support access to advanced functional testing, and reduction of inequities that arise from variability in institutional resources globally (Kernohan and Boycott, 2024). Equally critical is the development of interoperable, multi-institutional databases that securely integrate clinical, genomic and functional data, thereby enabling collaborative diagnostics, systematic variant reclassification, ongoing curation of gene–disease and variant–disease associations, and actionability options toward management and therapeutic interventions (Stenton et al., 2025; Mavraki et al., 2023). As the field moves toward a systems-level understanding of PMD, such biologically grounded and clinically accessible multidimensional diagnostics will be central to expeditiously ending the diagnostic odyssey and to delivering precision care that improves the survival, function and wellbeing of all affected patients with PMD.

## Supplementary Material

10.1242/dmm.052804_sup1 Supplementary information
