# Supplementary material for: Advances in diagnosis of mitochondrial dysfunction and primary mitochondrial disease
Source: Dis Model Mech. 2026 Oct 1;19(9):dmm052804. doi: 10.1242/dmm.052804 (PMC13670366; doi:10.1242/dmm.052804)
Supplement: Supplementary file 1 [file dmm-19-052804-s1.pdf]

Table S1. Genes associated with primary mitochondrial disease, grouped by functional class

Functional class	Subcategory	Genes	Inheritance
Homeostasis	Lipid metabolism / cardiolipin	*TAZ, AGK, PISD, SERAC1, TAMM41*	AR; X-linked
	Protein import	*AIFM1, GFER, DNAJC19, PAM16, PMPCA, TIMM8A, TIMM22, TIMM50, TOMM70, XPNPEP3*	AR; X-linked
	Ion / Ca2+ homeostasis	*LETM1, MICU1, MICU2*	AR
	Fission	*DNM1L, GDAP1, MFF, MIEF2, NME3*	AD or AR; AR
	Fusion	*MFN2, OPA1, SLC25A46, STAT2, YME1L1*	AD or AR; AR
	Processing / proteostasis	*HSPD1, CLPB, CLPP, HTRA2, LONP1, PITRM1, PMPCB, MIPEP, AFG3L2, SPG7, SACS*	AD or AR; AR
	Membrane / nucleoid architecture	*ATAD3A, C1QBP, CHCHD10, FBXL4, OPA3, TMEM65*	AD or AR; AD or de novo; AR
Cofactors	Coenzyme Q10	*COQ2, COQ4, COQ5, COQ6, COQ7, COQ8A, COQ8B, COQ9, HPDL, PDSS1, PDSS2, PYURF*	AR
	Iron-sulphur clusters	*BOLA3, FDX2, FDXR, FXN, GLRX5, HSCB, IBA57, ISCA1, ISCA2, ISCU, LYRM4, NFS1, NFU1, NUBPL, CIAO1*	AR
	Biotin	*BTD, HLCS*	AR
	Thiamine pyrophosphate	*SLC19A3, SLC25A19, TPK1*	AR
	Lipoic acid	*GCSH, LIAS, LIPT1, LIPT2, DLD, MCAT, MECR*	AR
	Heme	*COX10, COX15, PPOX, SLC25A38, CYCS, HCCS*	AD or AR; AD or de novo; AR; X-linked
	Copper	*SCO1, SCO2, COA6*	AR
	Coenzyme	*COASY, PANK2, SLC25A42*	AR
	S-adenosyl-methionine	*SLC25A26*	AR
	Riboflavin / FMN / FAD	*FLAD1, SLC25A32*	AR
	NADH	*NADK2, NAXD, NAXE*	AR
Inhibitors	Toxic metabolite accumulation / redox	*ETHE1, SQOR, IDH2, D2HGDH, L2HGDH, SLC25A1, ECHS1, HIBCH, HTT, TXN2, TXNIP*	AD or de novo; AR
Substrates	Substrate transport	*GOT2, MDH1, MPC1, SLC25A3, SLC25A10, SLC25A12, SLC25A13*	AR
	Pyruvate dehydrogenase complex	*PDHA1, PDHB, PDHX, PDP1, DLAT, PDK3*	AR; X-linked
	Anaplerosis	*PC, CA5A*	AR
	Beta-oxidation	*ACADM, ACADS, ACADSB, ACADVL, CPT1A, CPT2, HADH, HADHA, HADHB, SLC22A5, SLC25A20, ETFA, ETFB, ETFDH*	AR

	Ketone bodies	*ACAT1, HMGCL, HMGCS2, OXCT1*	AR
	Krebs cycle	*ACO2, DLST, FH, IDH3A, IDH3B, MDH2, OGDH, OGDHL, MRPS36*	AD or AR; AR
mtDNA maintenance and expression	mtDNA replication	*POLG, POLG2, TWNK, DNA2, MGME1, ENDOG, RNASEH1, SSBP1, TFAM, TOP3A, LIG3*	AD or AR; AR
	Nucleotide metabolism	*ABAT, DGUOK, DTYMK, GUK1, MPV17, RRM1, RRM2B, SAMHD1, SLC25A4, SUCLA2, SUCLG1, TK2, TYMP*	AD or AR; AR
	RNA processing and modification	*ELAC2, ERAL1, HSD17B10, MTPAP, LRPPRC, POLRMT, PRORP, PUS1, TEFM, THG1L, TRMT5, TRMT10C, TRNT1, TRMU, GTPBP3, MRM2, MTO1, NSUN3, PNPT1, TRIT1*	AD or AR; AR; X-linked
	Translation regulation	*GFM1, GFM2, GUF1, TSFM, TUFM, MTRFR, TACO1, RMND1*	AR
	Mitoribosomal subunits	*MRPL3, MRPL12, MRPL24, MRPL39, MRPL44, MRPL50, MRPS2, MRPS7, MRPS14, MRPS16, MRPS22, MRPS23, MRPS25, MRPS28, MRPS34, PTCD3, FASTKD2*	AR
	Mitochondrial aminoacyl-tRNA synthetases	*MTFMT, AARS2, CARS2, DARS2, EARS2, FARS2, GARS1, HARS2, KARS1, LARS2, IARS2, MARS2, NARS2, PARS2, QRSL1, RARS2, SARS2, TARS2, VARS2, WARS2, YARS2, GATB, GATC*	AD or AR; AR
	mtDNA-encoded tRNAs	***MT-TA, MT-TC, MT-TD, MT-TE, MT-TF, MT-TG, MT-TH, MT-TI, MT-TK, MT-TL1, MT-TL2, MT-TM, MT-TN, MT-TP, MT-TQ, MT-TR, MT-TS1, MT-TS2, MT-TT, MT-TV, MT-TW, MT-TY***	mtDNA
	mtDNA-encoded rRNA	***MT-RNR1***	mtDNA
OXPHOS subunits and assembly	Complex I subunits	*NDUFA1, NDUFA2, NDUFA6, NDUFA8, NDUFA9, NDUFA10, NDUFA11, NDUFA12, NDUFA13, NDUFB3, NDUFB8, NDUFB9, NDUFB10, NDUFB11, NDUFS1, NDUFS2, NDUFS3, NDUFS4, NDUFS6, NDUFS7, NDUFS8, NDUFV1, NDUFV2*	AR; X-linked
	Complex I assembly factors	*NDUFAF1, NDUFAF2, NDUFAF3, NDUFAF4, NDUFAF5 (C20orf7), NDUFAF6 (C8orf38), NDUFAF8 (C17orf89), NDUFC2, ACAD9, FOXRED1, TIMMDC1, TMEM126B, RTN4IP1, SFXN4, DNAJC30*	AR
	Complex I subunits (mtDNA-encoded)	***MT-ND1, MT-ND2, MT-ND3, MT-ND4, MT-ND4L, MT-ND5, MT-ND6***	mtDNA
	Complex II subunits and assembly factors	*SDHA, SDHB, SDHC, SDHD, SDHAF1, SDHAF2*	AD or AR; AR
	Complex III subunits and assembly factors	*CYC1, UQCRB, UQCRH, UQCRQ, UQCRC2, UQCRFS1, BCS1L, LYRM7, TTC19*	AR
	Complex III subunit (mtDNA-encoded)	***MT-CYB***	mtDNA
	Complex IV subunits and assembly factors	*COX4I2, COX5A, COX6A1, COX6A2, COX6B1, COX7B, COX8A, NDUFA4, SURF1, CEP89, COX11, COX14 (C12orf62), COX18, COX20 (FAM36A), COA3, COA5, COA7, COA8 (APOPT1), PET100, PET117*	AR; X-linked

	Complex IV subunits (mtDNA-encoded)	***MT-CO1, MT-CO2, MT-CO3***	mtDNA
	Complex V subunits and assembly factors	*ATP5F1A, ATP5F1B, ATP5F1D, ATP5F1E, ATP5MC3, ATP5PO, ATPAF2, TMEM70, ATP5MD, OXA1L, PPA2*	AD or AR; AD or de novo; AR
	Complex V subunits (mtDNA-encoded)	***MT-ATP6, MT-ATP8***	mtDNA

Mitochondrial DNA-encoded genes are shown in bold. Gene symbols follow HGNC-approved nomenclature. AD, autosomal dominant; AR, autosomal recessive; CoQ10, coenzyme Q10; FAD, flavin adenine dinucleotide; FMN, flavin mononucleotide; mtDNA, mitochondrial DNA; NADH, reduced nicotinamide adenine dinucleotide; OXPHOS, oxidative phosphorylation; rRNA, ribosomal RNA; tRNA, transfer RNA. Gene classification adapted from Heath et al. (2025)

Disease Models & Mechanisms • Supplementary information
